# Comparative Genomics of the Apicomplexan Parasites *Toxoplasma gondii* and *Neospora caninum*: Coccidia Differing in Host Range and Transmission Strategy

**DOI:** 10.1371/journal.ppat.1002567

**Published:** 2012-03-22

**Authors:** Adam James Reid, Sarah J. Vermont, James A. Cotton, David Harris, Grant A. Hill-Cawthorne, Stephanie Könen-Waisman, Sophia M. Latham, Tobias Mourier, Rebecca Norton, Michael A. Quail, Mandy Sanders, Dhanasekaran Shanmugam, Amandeep Sohal, James D. Wasmuth, Brian Brunk, Michael E. Grigg, Jonathan C. Howard, John Parkinson, David S. Roos, Alexander J. Trees, Matthew Berriman, Arnab Pain, Jonathan M. Wastling

**Affiliations:** 1 Wellcome Trust Sanger Institute, Hinxton, Cambridgshire, United Kingdom; 2 Institute of Infection and Global Health and School of Veterinary Science, Faculty of Health and Life Sciences, University of Liverpool, Liverpool, Merseyside, United Kingdom; 3 King Abdullah University of Science and Technology, Thuwal, Jeddah, Kingdom of Saudi Arabia; 4 Institute for Genetics, University of Cologne, Cologne, North Rhine-Westphalia, Germany; 5 Centre for GeoGenetics, Natural History Museum of Denmark, University of Copenhagen, Copenhagen, Denmark; 6 Department of Biology, University of Pennsylvania, Philadelphia, Pennsylvania, United States of America; 7 Program in Molecular Structure and Function, Hospital for Sick Children and Departments of Biochemistry and Molecular Genetics, University of Toronto, Toronto, Ontario, Canada; 8 Laboratory of Parasitic Diseases, National Institutes of Health, National Institute of Allergy and Infectious Diseases (NIAID), Bethesda, Maryland, United States of America; University of Georgia, United States of America

## Abstract

*Toxoplasma gondii* is a zoonotic protozoan parasite which infects nearly one third of the human population and is found in an extraordinary range of vertebrate hosts. Its epidemiology depends heavily on horizontal transmission, especially between rodents and its definitive host, the cat. *Neospora caninum* is a recently discovered close relative of *Toxoplasma*, whose definitive host is the dog. Both species are tissue-dwelling Coccidia and members of the phylum Apicomplexa; they share many common features, but *Neospora* neither infects humans nor shares the same wide host range as *Toxoplasma*, rather it shows a striking preference for highly efficient vertical transmission in cattle. These species therefore provide a remarkable opportunity to investigate mechanisms of host restriction, transmission strategies, virulence and zoonotic potential. We sequenced the genome of *N. caninum* and transcriptomes of the invasive stage of both species, undertaking an extensive comparative genomics and transcriptomics analysis. We estimate that these organisms diverged from their common ancestor around 28 million years ago and find that both genomes and gene expression are remarkably conserved. However, in *N. caninum* we identified an unexpected expansion of surface antigen gene families and the divergence of secreted virulence factors, including rhoptry kinases. Specifically we show that the rhoptry kinase *ROP18* is pseudogenised in *N. caninum* and that, as a possible consequence, *Neospora* is unable to phosphorylate host immunity-related GTPases, as *Toxoplasma* does. This defense strategy is thought to be key to virulence in *Toxoplasma*. We conclude that the ecological niches occupied by these species are influenced by a relatively small number of gene products which operate at the host-parasite interface and that the dominance of vertical transmission in *N. caninum* may be associated with the evolution of reduced virulence in this species.

## Author Summary

Coccidian parasites have a major impact on human and animal health world-wide and are among the most successful and widespread parasitic protozoa. They include *Neospora caninum* which is a leading cause of abortion in cattle and one of its nearest relatives, *Toxoplasma gondii*. Despite its close phylogenetic relationship to *Toxoplasma*, *Neospora* has a far more restricted host range, does not infect humans and its epidemiology depends predominantly on efficient vertical transmission. The divergent biology of these two closely related species provides a unique opportunity to study the mechanisms of host specificity, pathogenesis and zoonotic potential not only in these, but other Coccidia. We have sequenced the genome of *Neospora* and the transcriptomes of both species to show that despite diverging some 28 million years ago, both genome and gene expression remain remarkably conserved. Evolution has focused almost exclusively on molecules which control the interaction of the parasite with the host cell. We show that some secreted invasion-related proteins and surface genes which are known to control virulence and host cell interactions in *Toxoplasma* are dramatically altered in their expression and functionality in *Neospora* and propose that evolution of these genes may underpin the ecological niches inhabited by coccidian parasites.

## Introduction

*Toxoplasma gondii* and *Neospora caninum* are closely related tissue-dwelling Coccidia – intracellular protozoan parasites of the phylum Apicomplexa. *T. gondii* can infect essentially any warm-blooded vertebrate and is found in nearly one third of humans, arguably being the world's most successful zoonotic parasite [1]; it causes neonatal mortality, spontaneous abortion and blindness [2]. *T. gondii* is most often transmitted horizontally following ingestion of environmentally resistant oocysts excreted by its definitive host (cats), or via ingestion of persistent asexual stages (bradyzoites) residing in the tissues of intermediate hosts. The biology of *T. gondii* has been intensively studied, but despite advances in understanding host cell invasion, the role of secreted kinases in parasite virulence [3], [4] and its population and evolutionary biology [5], [6], the molecular mechanisms responsible for its highly promiscuous nature remain unknown.

*Neospora caninum* is a close relative of *T. gondii* [7]. They are both tissue-dwelling Coccidia and share many common morphological and biological features [8]. Each is able to develop in intermediate hosts, reproducing asexually, or to move between intermediate and definitive hosts, reproducing sexually. *Neospora* was initially misidentified as *Toxoplasma*, but was subsequently differentiated based on host preferences, etiology, morphological and genetic differences [8]. Despite these similarities the two species differ in their definitive host: while *Toxoplasma* completes its sexual cycle in felids, *Neospora* does so exclusively in canids [9]. Unlike *Toxoplasma*, *Neospora* appears not to be zoonotic, having a more restricted host range [10], [11] in which it occupies a unique ecological niche showing a striking capacity for highly efficient vertical transmission in bovines [12]. *N. caninum* is one of the leading causes of infectious bovine abortion, resulting in significant economic losses to the dairy and beef industries [13].

The molecular determinants of host specificity and in particular zoonotic capability in the Apicomplexa are not known. It is possible that a large part is played by the host cell invasion machinery common to all Apicomplexa which involves surface antigens and specialized apical secretory organelles named rhoptries, micronemes and dense granules [14], but this is yet to be substantiated by experimental evidence. The process of host cell invasion has been well studied in *Toxoplasma* and components of the invasion machinery are also involved in host cell modification and interaction with the host immune system [3], [4]. Attachment to host cells is mediated by a family of highly abundant surface antigens [15], after which the micronemes release adhesins which engage an actin-myosin motor to provide the driving force for host cell invasion [16], [17], [18]. Rhoptry neck proteins are then released to form a tight region of contact with the host cell, known as the *moving junction*, which acts as a scaffold for the parasite to enter the cell and form the parasitophorous vacuole (PV) in which it resides [19]. The rhoptries also release a range of proteins that modulate host cell function [20], [21], [22], in particular, virulence-related rhoptry kinases interact with host defenses; for example, ROP18 inactivates host immunity-related GTPases (IRGs) that would otherwise rupture the PV membrane and kill the parasite [23], [24].

Whilst it is anticipated that the overall process of host-parasite interaction in *Neospora* is likely to be similar, we hypothesize that the molecular characteristics of this interface are likely to be the key determinant in the unique biological features of the two parasites. In fact, small but defining differences in the biology of these two closely related organisms provide a unique opportunity to identify the mechanisms which underlie the basis of host specificity, pathogenesis and zoonotic potential not only in these important parasites, but also in the wider members of the phylum. This includes several groups of organisms of key interest to human and animal welfare (e.g. *Plasmodium*, *Cryptosporidium* and *Eimeria*).

In order to exploit this opportunity we have sequenced the genome of *N. caninum* and the transciptomes of both *N. caninum* and *T. gondii*, undertaking the first comparative transcriptome analysis of any apicomplexans at single base-pair resolution. We show that *Neospora caninum* and *Toxoplasma gondii* have very similar genomes with largely conserved gene content and synteny. As predicted, differences are most common amongst groups of genes which interact with the host. We find that surface antigen gene families are expanded in *N. caninum* suggesting that larger repertoires of such genes may be important in becoming a more host-restricted coccidian parasite, although data from a more extensive range of related parasites would be required to test this hypothesis. We also find that some rhoptry genes are highly variant between species and demonstrate that the pseudogenisation of *ROP18* in *N. caninum* leads to a functional change in the interaction of the parasite with host immune mechanisms. We propose that such mutations may be associated with changes in transmission strategy. In addition to these biological insights, our data provides a vital community resource for comparative genomics in this important phylum of medical and veterinary parasites.

## Results

### *Neospora caninum* genome sequence

**Figure 1:**
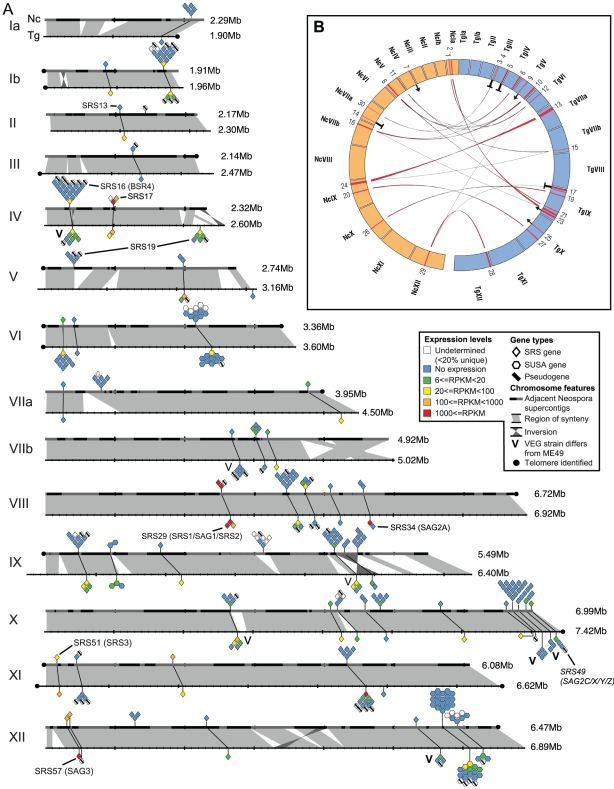
Chromosomal alignment of *N. caninum* Nc-Liv and *T. gondii* Me49 highlighting surface antigen gene families. (A) Aligned chromosomes of *N. caninum* (above) and *T. gondii* (below) showing conservation of synteny and distribution of SRS and SUSA surface antigen gene families. Tandemly repeated genes are shown clustered together. Uncoloured genes had less than 20% unique sequence and expression levels could not be accurately determined. 49 additional NcSRSs were found in UnAssigned Contigs (UACs), while three further TgSRSs were not assigned to chromosomes. (B) Shows putative rearrangements between *N. caninum* and *T. gondii* chromosomes. Large (>30 kb) insertions in one genome relative to the other are numbered on the chromosomes of *N. caninum* (orange) and *T. gondii* (blue). Red ribbons show regions of protein sequence similarity between these regions. The plot shows that most insertions have a pairwise relationship, e.g. region 13 from *T. gondii* chromosome VIIa is putatively orthologous to region 24 in *N. caninum* chromosome IX. Thus these regions are shared and not specific to one organism. The arrow symbol refers to sequence similarity with parts of the comparator genome not currently assigned to chromosomes (UACs). A capital ‘T’ identifies a region with no similarity in the comparator genome. These regions include genes belonging to novel families (TSF and KRUF).

We sequenced the genome of *N. caninum* Liverpool strain using Sanger sequencing to ∼8-fold depth. It was assembled into 585 supercontigs with an N50 of 359 kb totaling 61 Mb (Table 1). We constructed a set of *N. caninum* pseudochromosomes by aligning 242 supercontigs to the fourteen publicly available *T. gondii* Me49 chromosomes [25] based on predicted protein sequence similarity (Figure 1A). It has been shown previously using our partially assembled sequencing data that *N. caninum* and *T. gondii* genomes are largely syntenic [26]. Here we show that for almost all regions where conservation of gene order (synteny) is interrupted, corresponding orthologous regions are found elsewhere in the genome. This suggests that while there may have been a small number of chromosomal rearrangements, there has been very little net gain or loss of genetic content (Figure 1B). The *N. caninum* Liverpool genome sequence has been added to the European Nucleotide Archive as project CADU00000000.

**Table 1:** Composition of *N. caninum* genome in comparison with *T. gondii* and *P. falciparum*.

	*Neospora caninum* NcLiv	*Toxoplasma gondii* Me49	*Plasmodium falciparum* 3D7
**Genome size (Mb)**	61.0	63.0	23.3
**Chromosomes**	14	14	14
**G+C content (%)**	54.8	52.3	19.4
**Protein coding genes**	7121	7286 (7993)	5383
**Mean gene length (bp)** *	2553	2341 (2236)	2292
**Gene density (genes per kb)**	0.116	0.121 (0.126)	0.231
**Percent of genome encoding proteins**	29.7	28.3	53.0

*T. gondii* gene models were downloaded from ToxoDb v5.2 [25]. Values for *T. gondii* were calculated after manual curation, those in brackets are the original values based on ToxoDb v5.2. *P. falciparum* data were obtained from the February 2010 release of GeneDB [94].

*: - Excluding introns and UTRs.

### Transcriptome sequencing of *N. caninum* and *T. gondii*

To determine gene expression differences between species and to improve genome annotation we sequenced the transcriptome of the invasive stage (tachyzoite) of *N. caninum* Liverpool and *T. gondii* VEG using mRNA sequencing (mRNAseq) on an Illumina GAIIx machine (Tables S5 & S6). The parasites were grown asynchronously for a period of six days in cell culture. We took samples of RNA at days three, four and six. We found that days three and four showed fairly similar expression profiles within species and so we have pooled this data for most analyses. We found however that day six *N. caninum* parasites were showing expression of bradyzoite (quiescent stage) marker genes (Text S1). These parasites had not fully converted into bradyzoites, but may be preparing to do so. We did not observe expression of these markers at day six in *T. gondii*, so we did not seek to compare transcriptomes of the two species at this timepoint. Transcriptome sequencing data has been submitted to ArrayExpress with accessions E-MTAB-549 for *N. caninum* sequences and E-MTAB-550 for *T. gondii* sequences.

### Gene content is largely conserved between species

Combining *de novo* gene predictors and mRNAseq evidence we identified 7121 protein-coding genes in *N. caninum* and produced a revised *T. gondii* ME49 gene count of 7286, a reduction of 9% from previous predictions (Table 1). This was achieved predominantly by merging adjacent genes based on mRNAseq evidence. In *N. caninum* we detected the expression of 74% of genes during the tachyzoite stage. In *T. gondii* 80% were expressed, significantly more than the 49% recently reported using microarrays suggesting greatly improved sensitivity [27].

**Figure 2:**
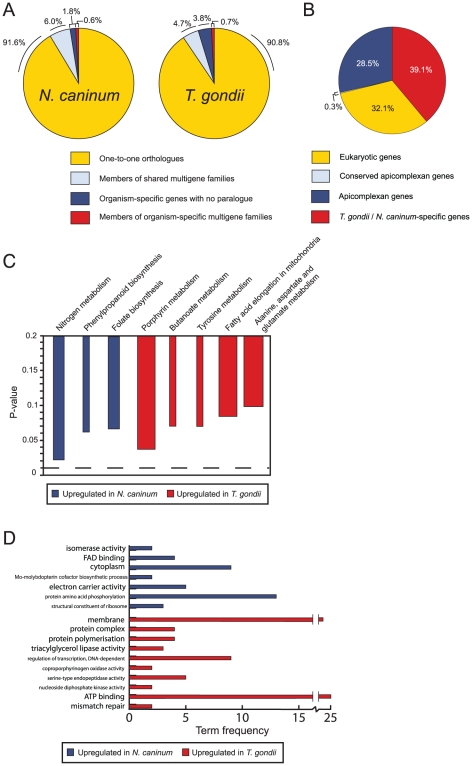
Protein-coding gene content and metabolic activity are largely conserved between the two species. (A) Most protein-coding genes in *N. caninum* have a one-to-one orthologous relationship (yellow) with a gene of *T. gondii*. A larger proportion of the *T. gondii* genome consists of genes with no *N. caninum* homologue than vice versa (organism-specific genes in red). The increase in shared multi-gene families (blue) in *N. caninum* reflects the expansion of SRS genes in this organism. The increase in organism-specific multigene families (red) in *T. gondii* reflects, for instance, the TSF gene family identified by us in this work. (B) Of the one-to-one orthologues shared by *T. gondii* and *N. caninum* we identified those which have orthologues in three or more non-apicomplexan eukaryotes (yellow), are not present in three or more apicomplexans but in all apicomplexan groups sequenced to date (grey), are in at least one other apicomplexan group (blue) or are specific to *T. gondii* and *N. caninum* (red). (C) Pooled day three and four RNAseq experiments were used to determine orthologous genes differentially expressed between *T. gondii* and *N. caninum*. Differentially expressed genes were examined for enrichment with enzymes from different KEGG pathways as described in methods. No pathways were identified with a p-value less than 0.01, although putative differences were identified at a p-value cutoff of 0.05. The width of bars in the chart relates to the number of genes in the pathway which were differentially expressed, e.g. *Porphyrin metabolism* had three enzymes differentially expressed, while *Nitrogen metabolism* had two and *Tyrosine metabolism* one. Only pathways with a p-value (adjusted for multiple hypothesis testing) below 0.1 are shown. (D) Gene Ontology terms over represented amongst genes upregulated in *N. caninum* and *T. gondii*. All terms shown are significantly upregulated (P<0.05). The terms ‘membrane’, ‘regulation of transcription, DNA-dependent’ and ‘ATP binding’ are found more often than expected in genes upregulated in *T. gondii*. SRS surface antigens, rhoptry kinases and AP2 transcription factors respectively are associated with these terms, suggesting that SRSs, ROPs and AP2s are amongst the most highly upregulated groups of genes in *T. gondii* relative to *N. caninum*. The term ‘protein amino acid phosphorylation’ is overrepresented amongst genes upregulated in *N. caninum* relative to *T. gondii*. Many of the genes in this group are rhoptry kinases suggesting that while some are upregulated in *T. gondii*, others are upregulated in *N. caninum*. These findings are explored in more detail in Figure 3.

Using a combination of automated orthologue identification and manual curation we identified a small number of unique (i.e. organism-specific) genes in both genomes that might underlie their phenotypic differences (Figure 2A). Excluding surface antigen families (discussed later), we found 231 genes unique to *T. gondii* and 113 to *N. caninum*, i.e. with no orthologue or paralogue based on our orthologue analysis. Of these, 72 from *T. gondii* and 43 from *N. caninum* had Pfam domains or proteomics-based evidence (Table S1). These genes represent good candidates for understanding organism-specific differences and are enriched for those involved in host-parasite interactions. The remainder had no detectable homologues or proteomics-based evidence, although most had good transcriptome evidence.

Only one organism-specific multigene family, with no homologues in the other species was identified: a family that we have named Toxoplasma-specific family (*TSF*; Figure S1). This family is located largely in chromosomal regions with no similarity to *N. caninum* (regions 3, 5 and 17 in Figure 1B) and varies in size between *T. gondii* strains. We found that all ten members of *TSF* from *T. gondii* Me49 were expressed during the tachyzoite stage. No significant domains, motifs or signal peptides were identified; however a putative transmembrane helix was predicted between amino acids 195 and 217 on TgTSF1.

Another previously unidentified family was present in *N. caninum*, but was expanded in *T. gondii* (Figure S2). This family comprises three genes from *N. caninum* and seven from *T. gondii* Me49. Sequences were scanned using InterProScan [28] but no significant domains or motifs were identified. Lysine-arginine rich motifs are however present in the sequences suggesting possible nuclear localization signals. We have therefore named this family Lysine-Arginine rich Unidentified Function (KRUF). KRUF genes appear to be highly expanded in the GT1 strain of *T. gondii*, with up to twenty members [25]. Two of the three *N. caninum* members are expressed in the tachyzoite and early bradyzoite stages (NCLIV_002020 and NCLIV_002030). Most of the *T. gondii* members are expressed, some at very high levels (esp. TGME49_051170).

While 32% of the genes shared by *T. gondii* and *N. caninum* have orthologues in a range of eukaryotes, we found that ∼39% of the shared genes do not have orthologues in other apicomplexans sequenced to date (Figure 2B). Furthermore, while ∼29% of the shared genes not found outside apicomplexans have orthologues in at least one apicomplexan, only 0.3% are shared between all apicomplexans with completed genome sequences. Due to the assumptions behind this analysis we have likely underestimated the similarity between Apicomplexa and more detailed manual analysis will no doubt reveal more divergent orthologues. However our results suggest that the genome content of apicomplexans is very diverse and that many novel and divergent genes are found within the Coccidia.

### Metabolic capacity is conserved between species

To determine whether their divergent lifestyles are associated with differences in metabolism we compared the predicted repertoires of metabolic enzymes and pathways of *N. caninum* to those of *T. gondii* [25]. The pathways identified in *N. caninum* appeared identical to those in *T. gondii* and we found no single metabolic gene specific to either species suggesting that changes in metabolism do not play a large role in host restriction and zoonotic compatibility in these species. Although a small number of metabolic genes were differentially expressed between species, we found little evidence that these were clustered in any particular pathway, although there is some evidence that nitrogen metabolism may be upregulated in *N. caninum* and porphyrin metabolism may be upregulated in *T. gondii* (Figure 2C).

### *T. gondii* and *N. caninum* diverged from their common ancestor around 28 million years ago

Previous estimates from rRNA analysis have suggested that *N. caninum* and *T. gondii* diverged between 12 and 80 million years ago (mya) [6], [29]. To gain a more accurate estimate we examined a large number of orthologue alignments, determining synonymous substitution rates between *N. caninum* and *T. gondii* and between malarial parasites of human and non-human homonidae: *Plasmodium falciparum* and *P. reichenowi*, respectively. We assumed constant evolutionary rates between the *Plasmodium* spp. and Coccidia, excluding genes which were found to have evolved in a non-clock-like manner. We used a previously determined estimate of 2.49 mya for the split between *P. falciparum* and *P. reichenowi* [30]. This allowed us to date the speciation of *N. caninum* and *T. gondii* to 28.0 mya or between 21.7 and 42.7 mya using the confidence intervals of the *P. falciparum* and *P. reichenowi* divergence time. This suggests that speciation of *N. caninum* and *T. gondii* occurred after the speciation of their definitive hosts (estimated at 54–67 mya) [31]. The ability to reject non-clock-like genes is dependent on gene length and so we also calculated the divergence time using only the longest 25% of the orthologous groups. This led to a divergence time of 26.9 mya, very close to that calculated using all groups, suggesting that a tendency to exclude longer genes using the clock test has not biased our results.

### Surface antigen gene families are greatly expanded in *N. caninum*

**Figure 3:**
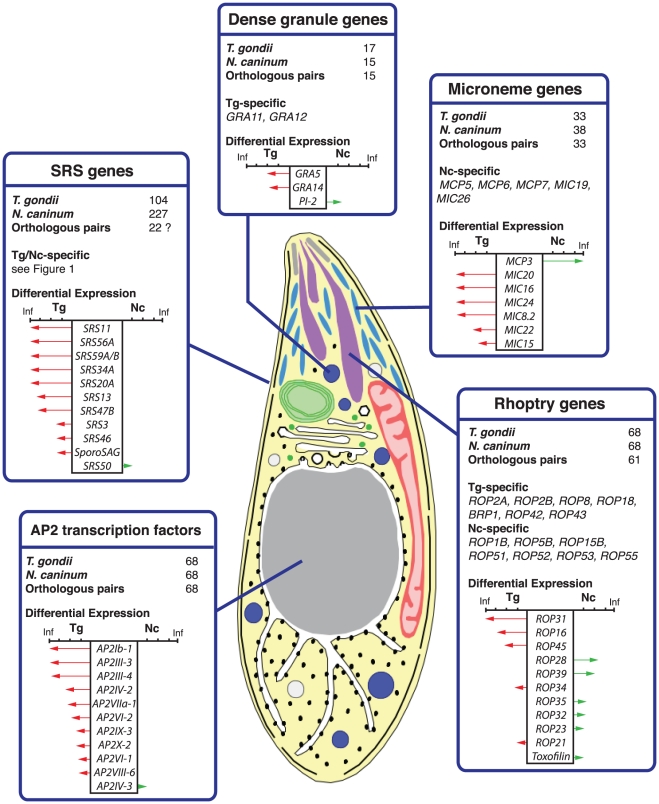
Repertoires and differential expression of known and predicted host-interaction genes and AP2 transcription factors. It was only possible to reliably identify orthologues for 22 SRS genes due to the way they have expanded, often being in large tandem arrays subject to gene conversion. While the AP2 transcription factors are not directly involved in host-parasite interaction they may be important in regulating expression of invasion genes. Each report card details the comparative repertoires of a particular group of genes in these species, the names of the genes specific to each organism and those which are differentially expressed between organisms. Further details of these relationships, including reference numbers, are included in Table S4. Arrows show the fold change in expression (RPKM; Reads Per Kilobase per Million mapped reads) between the two species on a log_2_ scale. The ticks are 2, 6 and 8 on this scale. Green arrows highlight increased expression in *N. caninum* tachyzoites. Red arrows highlight genes with increased expression in *T. gondii* tachyzoites vs. *N. caninum* tachyzoites. A fold change is infinite where the gene is not expressed at all in one organism.

Examination of gene gain and loss and differential expression implicated two host-interaction gene families: SAG1-Related Sequence (*SRS*) and *ROPK*, as among the most divergent features of the two species (Figure 2D, Figure 3). SAG1 was the first SRS protein identified and is the major surface antigen of *Toxoplasma*. SRS proteins localize to the cell surface of both *T. gondii* and *N. caninum*. They are thought to play a key role in attachment to host cells, modulation of host immunity and regulation of parasite virulence [32]. Wasmuth et al. (submitted) found 109 functional genes and 35 pseudogenes in *T. gondii* Me49 with similar numbers across several different strains. They are present sometimes in single copies, often in tandem arrays. They are dispersed across all chromosomes rather than showing a preference for subtelomeric regions as is found for some large gene families in *Plasmodium*, *Babesia* and *Theileria* (Figure 1A). It has been suggested that the large number of SRS genes is present in *T. gondii* to accommodate the wide spectrum of potential host-cell molecular interactions presented by its exceptionally large host range [33], [34]. However, our data refute this; we found the *SRS* gene family to be substantially expanded in *N. caninum* compared to *T. gondii* with a total of 227 *N. caninum SRS* genes (*NcSRSs*) and 52 *NcSRS* pseudogenes (Figure 1A). Expression data suggest however that *T. gondii* expresses a greater number of its *SRS* repertoire (55 vs. 25 in *N. caninum*) during the tachyzoite stage (Figure 1A). In *N. caninum* we found in most cases that only a single *SRS* gene was expressed at a multigene locus, whereas in *T. gondii* we often found several. Extending our gene expression studies beyond the rapidly growing and invasive tachyzoite stage, we noticed that *N. caninum* cultures maintained until day six showed expression of known bradyzoite-specific genes (e.g. *BAG1*, *SRS13*, *SAG4*), suggesting they were beginning to convert into the slow-growing stage (Text S1). We observed a greater number of *NcSRS* genes (36 vs. 25) expressed at day six than at earlier points in the culture. Despite this it remains unclear whether most members of this expanded family in *N. caninum* are expressed and further expression data are required from all life-stages before the role of these genes can be better understood.

*SRS* genes consist of one or more copies of the SAG domain family, which has been classified into eight subfamilies (Figure S3; Wasmuth et al., submitted). The doubling of *SRS* gene numbers in *N. caninum* compared to *T. gondii* is largely accounted for by expansion of a particular subfamily with a 7–8 domain architecture. No novel SAG domain subfamily has evolved in either lineage, however several domain combinations are found in low copy numbers in only one or the other species (Table S2). Since a particular *SRS* locus tends to contain genes with the same domain architecture in both species, expansion has likely occurred by tandem duplication. We found evidence that gene conversion may have occurred at, at least, one locus (*SRS19*; Figure S4A), whereas one of the most highly expressed loci in both organisms (*SRS29*, containing the *SAG1* gene) showed no evidence of gene conversion (Figure S4B), perhaps due to functional constraints.

*SUSA* genes (SAG-Unrelated Surface Antigen genes) are a superfamily of surface antigens unrelated to *SRSs* but which are also postulated to interact with the host immune system [35]. In common with the *SRS* superfamily we found that *N. caninum* had an expanded number of *SUSA* genes but that a greater number were expressed in *T. gondii* (Figure 1A). In fact none of the *NcSUSA* genes were expressed in the tachyzoite stage. Two *NcSUSA* genes (NCLIV_067570 and NCLIV_067920) were however expressed at day six of culture.

### Variation in rhoptry kinase genes highlights evolution of host-parasite interaction

The apical complex is the defining characteristic of the Apicomplexa. It includes the rhoptry, microneme and dense granule secretory organelles, which are essential for cell invasion. Figure 3 shows how the repertoires and expression of gene products known or predicted to be localized to these organelles differs between *T. gondii* and *N. caninum*. Several *T. gondii* rhoptry genes (*ROP18*, *ROP16* and *ROP5*) have been implicated in virulence based on a genetic cross between the type II and III [3] and type I and III [4] lineages of *T. gondii*. *N. caninum* differs from *T. gondii* at each of these loci, but shares some similarities with low virulence strains.

### Pseudogenisation of ROP18 in *N. caninum* prevents ROP18-mediated inactivation of immunity-related GTPases

**Figure 4:**
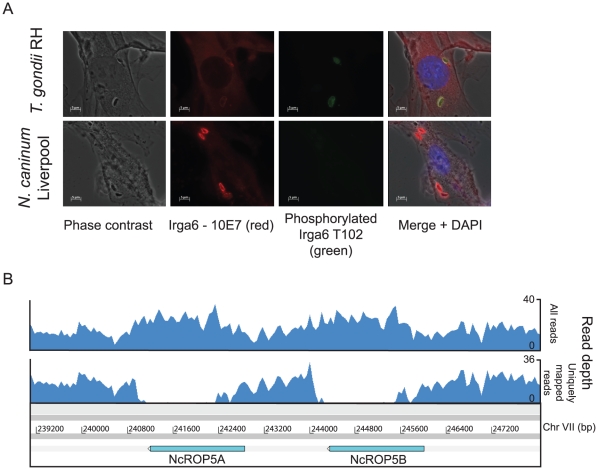
ROP18-mediated IRG inactivation is absent in *N. caninum* and *ROP5* has a lower copy number in *N. caninum* than *T. gondii*. (A) Phosphorylation of Irga6 T102 is observed in Mouse Embryonic Fibroblasts (MEFs) infected with *T. gondii RH* (a virulent strain) but not in *N. caninum NcLiv*-infected MEFs despite loading of Irga6 onto the PV. (B) Genomic Illumina sequencing reads are shown mapped to a reassembled ROP5 locus in *N. caninum*. This sequencing was performed using a PCR-free protocol to remove biases commonly introduced by PCR, resulting in more even coverage. The SSAHA mapping algorithm maps reads to multiple locations if they map equally well (mutireads), but gives these reads a mapping quality score of 0. The upper plot shows all mapping reads (including multireads) and slightly elevated read depth is visible over most of each ROP5 gene. Removing reads mapping with a mapping quality of less than ten shows that reads cannot be mapped reliably to most of each gene because they are almost identical in sequence. Therefore, allowing all reads to map, we would expect an average doubling of read depth over these genes if there were really only two copies and an increase in read depth of four times above background if there were four copies. We conclude that there are only two copies of the ROP5 gene, as in the original assembly.

Expression of *TgROP18* is associated with virulence in mice [4] and in some hosts high ROP18 expression may reduce parasite fitness by causing rapid host death [36]. It is involved in preventing the host interferon-gamma (IFN-γ) response, during which the host loads immunity-related GTPases (IRGs) onto the parasitophorous vacuole (PV) leading to its disruption and parasite cell death in avirulent strains [37]. Virulent *T. gondii* strains express high levels of ROP18, which phosphorylates and inactivates IRGs to safeguard the PV [24], [37]. We found that in *Neospora* Nc-Liv *ROP18* is a pseudogene due to several interrupting stop codons in the sequence syntenic with the *Toxoplasma* gene. We confirmed the presence of these stop codons in a further four strains of the parasite isolated from different geographic locations and hosts (Table S3). To determine whether *N. caninum* is able to phosphorylate IRGs without a functional copy of *ROP18* we examined the loading of Irga6 (a member of the host IRG GTPase family) onto the PV by immunofluorescence studies. We observed that, in both *N. caninum* and *T. gondii* infections, host cells responded by loading Irga6 onto the PV but only *T. gondii* was able to phosphorylate Irga6 and thereby presumably inactivate the IRG protein (Figure 4A). This suggests that *N. caninum* is unable to prevent its host from using IRGs to attack the PV.

### *ROP16* and other virulence-associated rhoptry kinases

In *T. gondii* ROP16 directly interferes with host signaling pathways (e.g. Stat3, Stat6) to modulate the proinflammatory host cytokine IL-12 [21], [38]. A single polymorphic residue on TgROP16 determines the strain-specific activation and phosphorylation of Stat3 [39]. We found that *ROP16* was highly expressed in *T. gondii* VEG (type III) tachyzoites and it has been shown elsewhere to be highly expressed in all *T. gondii* strain types [27]. While its orthologue in *N. caninum*, *NcROP16*, possesses the key active-site leucine residue for Stat3 activation, the gene was not expressed in our experiments. Although it is possible that *NcROP16* could be expressed in other cell types, our experiments predict that *N. caninum* infection does not activate Stat3 due to its lack of expression. Several additional *T. gondii* rhoptry genes are missing from *N. caninum* (Figure 3), most notably the entirety of the locus which encodes *ROP2A*, *ROP2B* and *ROP8*. The *TgROP5* multigene locus accounts for 50% of inherited variation in *Toxoplasma* virulence [40]. The relationship between *ROP5* genotype and virulence in *T. gondii* is however not clear. The most virulent, type I *T. gondii* strain (e.g. RH) has six copies, while the less virulent type II *T. gondii* strain (e.g. Me49) has around ten copies and the least virulent type III strain (e.g. VEG) has four. *N. caninum* Liverpool encodes only two copies of the *ROP5* gene both of which are highly expressed in the tachyzoite stage (Figure 4B).

### Divergence among other apical organelllar genes

The secreted proteins of the microneme organelles play a crucial role in host cell attachment and invasion by mediating gliding motility [16]. We identified thirteen previously undescribed genes putatively encoding micronemal proteins by virtue of conserved domain architectures. Of these newly identified genes, *MIC26* (a *MIC2* paralogue) and *MIC19* (a PAN domain-containing gene) are unique to *N. caninum*. Some differences also exist between the species in dense granule genes which are involved in the modification and function of the parasitophorous vacuole (PV) [41]. Dense granule genes *GRA11* and *GRA12* were absent from the *N. caninum* genome sequence.

Serine proteases are important to the maturation of both rhoptry and microneme proteins and their inhibition blocks parasite replication and rhoptry formation [42]. TgSUB2, a subtilisin-like serine protease has been identified as a likely processor of several rhoptry proteins [43] and whilst *T. gondii* is vulnerable to a variety of protease inhibitors, including serine protease inhibitors, *N. caninum* invasion is inhibited only by aspartyl protease inhibitors [44]. We found that while all 12 identifiable *T. gondii* subtilases had orthologues in *N. caninum*, there was a significant decrease in expression of these proteases (hypergeometric test; p = 0.003) compared with *T. gondii*. This suggests that subtilisin-like serine protease activity may not be used to the same extent in *N. caninum* as in *T. gondii* and may explain why *N. caninum* is less susceptible to its inhibition.

### Evolution of transcriptional regulation

The *ApiAP2* family represents the major group of apicomplexan transcription factors. They have been implicated, for example, in control of the intraerythrocytic development cycle (IDC) [45] and sporozoite development [46] of malaria parasites. Twenty-nine such genes have been identified in *Plasmodium* and 68 in *Toxoplasma* [47]. We found *N. caninum* orthologues for all 68 *TgAP2* genes but detected significant differences in the expression of eleven of them (Figure 3), which in turn may be responsible for expression differences we have observed in other genes. It has been suggested for instance that rhoptry genes are regulated by AP2 transcription factors in *Plasmodium* [48]. We found that 54 of 68 *NcAP2s* and 61 of 68 *TgAP2s* were expressed during the tachyzoite stage, more than a previous study [49]. This is surprising considering that one would expect the principal family of transcription factors in organisms with a complex life cycle to be highly specific to different life stages.

As expected, the repertoire of ncRNA genes of known function (e.g. t-RNAs, snoRNAs, snRNAs etc.) is almost identical between *Toxoplasma* and *Neospora*. However, we were able to identify an expansion of a previously unidentified candidate structured non-coding RNA family in *N. caninum*. This suggests that ncRNA repertoires are divergent in these species, although the functions of these RNAs remain to be identified (Text S2, Figures S5 & S6).

## Discussion

We have used genome and transcriptome sequencing to probe the apicomplexan parasites *Toxoplasma gondii* and *Neospora caninum* for differences which might underlie their divergent host ranges, transmission strategies and zoonotic potential. We have demonstrated that the two genomes show a high degree of synteny, with a one-to-one correspondence between most protein-coding genes. We calculated that speciation occurred around 28 mya, after the divergence of their respective definitive hosts, the cat and dog. This is consistent with two possibilities: 1) one or both parasite species may have switched to a new definitive host since their divergence, 2) a common ancestor used both cats and dogs as definitive hosts but during divergence *N. caninum* and *T. gondii* eventually became restricted to their present day definitive hosts.

Our data clearly show that genes interacting most closely with the host have diverged to the greatest extent and we have therefore been able to narrow investigations to a relatively small number of candidate gene families and individual genes. Although many genes of unknown function remain to be characterized in these organisms, the majority of these are conserved. We have identified two novel protein-coding gene families (TSF and KRUF) and a putative ncRNA family which differs between species and that warrant further experimental characterization.

The principle surface antigen gene family, the SRSs, was the most divergent family. This result was expected because in all Apicomplexa examined so far, including several malaria parasite genomes, the surface antigens are the largest, most rapidly evolving of all gene families [50]. However, the observation that *N. caninum* has more than twice as many SRS genes as *T. gondii* is striking and rather unexpected. It had been assumed that *T. gondii* requires a large number of these genes to accommodate its extraordinarily large host range and cover all potential host-cell molecular interactions with corresponding parasite proteins [33], [34]. The much smaller host range of *N. caninum* would suggest that this hypothesis is not supported and that perhaps conversely, a larger number of SRS genes might be advantageous in evolving a narrower host range. Transcriptome evidence however suggests that *N. caninum* uses fewer SRSs than *T. gondii* during the tachyzoite stage, suggesting that they may be of more importance in other parts of the life cycle. In fact, in *N. caninum* there is rarely more than one SRS gene expressed at each locus, while in *T. gondii* there are frequently multiple genes expressed. This implies there have been significant changes in regulation of these host-interacting genes between species, although the mechanisms of regulation of these genes remain unknown. Interestingly it is a small number of subfamilies which have been expanded in *N. caninum*, in particular the fam7-8 architecture, the most common in both species. It may be that the more limited host range of *N. caninum* is related to specialization of this subset of the SRS genes.

In common with SRS genes, important species-specific differences were identified in rhoptry organelle genes where the divergence of key genes of known function may help to explain phenotypic differences between *Toxoplasma* and *Neospora*. In particular ROP18 is a key virulence determinant in *T. gondii* which protects the parasitiphorous vacuole from attack by the mouse immune system [23]. We showed that this gene is pseudogenised in *N. caninum* and that *N. caninum* is unable to perform ROP18-mediated inactivation of immunity-related GTPases (IRGs) in murine cells.

Our data suggest a reduced role for *T. gondii* virulence factor orthologues in *N. caninum*, for example, in relation to the virulence-associated rhoptry proteins ROP18, ROP16 and ROP5. The loss of ROP18 function in *N. caninum* might be adaptive, preventing killing of its host and promoting parasite survival in the species to which it is restricted. Intriguingly, it has been proposed and shown experimentally in viruses that reduced virulence is associated with the evolution of vertical transmission [51], [52], one of the most striking characteristics of *N. caninum* transmission in cattle. *N. caninum* may have increased successful vertical transmission from cow to calf by reducing virulence mechanisms, thus reducing the likelihood of host mortality. Alternatively, if ROP18 is only relevant to a subset of *T. gondii* intermediate hosts, its loss in *N. caninum* may reflect the fact that these intermediate host species are less important to *N. caninum*. Indeed, the importance of the cat-mouse cycle in the epidemiology of *T. gondii* may explain the evolution of ROP18-mediated inactivation specifically of murine IRGs, a mechanism which is certainly less relevant to *N. caninum* in which canids rather than felids are the definitive host. In fact IRG homologues are known to be present in the bovine genome [53] and novel *N. caninum* rhoptry genes could mediate IRG-defense in these hosts. Both of these scenarios suppose that *N. caninum* has become more specialized in its host range, suggesting that the common ancestor of *N. caninum* and *T. gondii* had a wide host range. In order to test this hypothesis it will be necessary to examine the genomes of coccidian outgroups such as *Sarcocystis* and *Eimeria* and to better characterize the function of those rhoptry proteins specific to *N. caninum*.

The genomic resource we present will be useful in generating further understanding of apicomplexan genome evolution in general and coccidian biology in particular. Furthermore our description of these parasites will help to kick-start large-scale population-based studies to understand how genetic variation affects their biology.

## Materials and Methods

### Parasite cultivation

*Neospora caninum* Liverpool strain was originally isolated from the cerebrum of a congenitally infected dog [54]. *N. caninum* Liverpool and *Toxoplasma gondii* VEG tachyzoites were maintained as described previously [55].

### Genome sequencing and assembly

Paired end reads of *N. caninum* DNA were generated from random subclone libraries and additional reads were directed to close gaps and improve the data coverage of low quality regions. All sequencing was performed using BigDye terminator chemistry and used AB 3730xl analyzers (Life Technologies). In total, 920k reads were obtained, quality-clipped and screened for contamination. 92% of reads were used in the final assembly. Based on an estimated genome size of 62 Mb for *N. caninum* the sequencing coverage was ∼8x. Sequence reads were assembled using PHRAP (P. Green, unpublished) into 960 supercontigs with an N50 of 354 kb. To reanalyse the ROP5 locus these reads were reassembled using Arachne [56].

*N. caninum* pseudochromosomes were generated by aligning supercontigs to *T. gondii* ME49 chromosomes using PROmer [57]. 242 contigs (90.4% of the sequence) aligned successfully to the 14 chromosomes of *T. gondii*. Of the remaining 718 contigs, 375 were removed due to contamination, poor quality or if they were <1 kb in length. The remaining 343 contigs were grouped as UnAssigned Contigs (UACs) and used in further analysis alongside the pseudochromosomes. Telomeres were identified by examining chromosome ends for the typical TTTAGGG septameric repeat.

A genome resequencing library for *N. caninum* were prepared as in [58]. Sequencing was performed on an Illumina GAIIX as for transcriptome libraries. Illumina paired-end reads were mapped using SSAHA2 [59].

The ROP18 region of five *N. caninum* isolates (Table S3) was amplified in two overlapping sections using the following primer pairs: F1+R3 and F3+R1 (exp. product 1268 bp and 890 bp respectively) (supplied by Eurofins). ROP18_F1 – GAGTGCCACGGTCCTCTAAG, ROP18_R3 – ATTTGTCCGACGCAAAATTC, ROP18_F3 – GGCTTCTGCTCCAGTATTCG, ROP18_R1 – GCCTTATAAACCACCCGTCA. PCR Reagents were supplied by Qiagen.

*Toxoplasma* genome sequences and gene models were downloaded from ToxoDb v5.2 (http://www.toxodb.org); they were generated at the J. Craig Venter Institute and have been kindly provided by the *Toxoplasma* research community.

### Gene finding and annotation

*N. caninum* gene models were created using several algorithms [60]–[66] trained on *T. gondii* Me49 (ToxoDB v4.2) and using ESTs from *N. caninum* Liverpool and NC-1 strains collected by the Gene Index Project [67]. The models were examined using the Artemis Comparison Tool [68] and where possible corrected based on evidence from synteny and sequence conservation with *T. gondii* and transcriptome sequencing evidence. We found a large number of erroneously unfused gene models. Using our *T. gondii* transcriptome data a total of 354 pairs of adjacent, same-strand genes were linked by reliably mapped bridging read-pairs. A further 449 genes in *T. gondii* were found to be parts of an adjacent gene but did not have spanning read pairs, usually being likely UTR segments. This resulted in a large drop in the predicted *T. gondii* gene count and we incorporated these corrections into our subsequent analysis.

We used orthoMCL [69] to identify an preliminary set of orthologous groups between *T. gondii* and *N. caninum*. These results was modified using 679 manually identified orthologue pairs. We identified 6348 one-to-one orthologous gene pairs, which we then used to determine whether genes in these organisms tend to be shared with other apicomplexan species. We performed an orthoMCL with representative genes for the one-to-one (core) *T. gondii*/*N. caninum* set as well as predicted protein sequences for the following species: the plant *Arabidopsis thaliana*, the piroplasmic apicomplexan *Babesia bovis*, the apicomplexan *Cryptosporidium parvum*, the slime-mould *Dictyostelium discoidium*, human, the haemosporian apicomplexan *Plasmodium falciparum*, the yeast *Saccharomyces cerevisiae*, the piroplasmic apicomplexan *Theileria annulata*, the kinetoplastid *Trypanosoma brucei* and the diatom *Thalassiosira pseudonana*. Where a core gene was found to have an orthologue in three or more non-apicomplexan eukaryotes, we defined it as *eukaryotic*. Where it was not eukaryotic, but conserved amongst all apicomplexa, we defined it as *conserved apicomplexan*, If a gene was not *conserved apicomplexan*, but found in one or more apicomplexan species, other than *T. gondii* and *N. caninum* it was defined as *apicomplexan*. Remaining genes were considered specific to *T. gondii*/*N. caninum*.

SRS genes and SAG domains were identified as in Wasmuth et al. (submitted). We identified pseudogenes as clusters of significant BLAST hits which did not overlap valid gene models. Putative pseudogenes were manually checked to determine whether rational gene models could be made and whether Ilumina resequencing data supported any stop codons. *N. caninum* SAG domains were clustered as in Wasmuth et al., to identify any novel domain subfamilies.

*N. caninum ROP*, *MIC*, *GRA* and *AP2* genes were initially determined by manually identifying orthologues of known *T. gondii* genes with reference to various studies [70]–[77]. Where homologous families of proteins fell into these groups, e.g. ROPK family for *ROP*, TRAP and MAR for *MIC*, novel members were sought using BLAST and HMMer.

### Transcriptome sequencing and mapping

Poly A+ mRNA was purified from total RNA using oligo-dT dyna bead selection followed by metal ion hydrolysis fragmentation with the Ambion RNA fragmentation kit. 1st strand cDNA was synthesized using randomly primed oligos followed by 2nd strand synthesis to produce dscDNA. Fragments were selected for 200–250 bp inserts amplified by PCR to enrich for properly ligated template strands. Libraries were sequenced using the Illumina Genome Analyzer IIX in paired end mode for 2×76 cycles using proprietary reagents according to the manufacturer's recommended protocol.

RNA-seq reads were aligned against the reference genomes using SSAHA2 [59]. Reads were included only where one end of the pair aligned uniquely to the genome and the distance between the pairs was within the expected insert size range, plus the expected intron length (80–4000 bp).

### Expression analysis

We used Reads Per Kilobase of exon model per Million mapped reads (RPKM) normalised by the unique length of the gene as a measurement of expression level. We excluded positions which were non-unique from the length calculation using a kmer window of 75 bp, 37 bp either side of that position. Non-uniquely mapped reads were excluded by removing reads with a score <10. In order to determine whether or not a gene was expressed we calculated an RPKM threshold (Figure S7).

We used DESeq to determine differentially expressed genes [78]. In each pairwise comparison of two conditions A and B (e.g. *N. caninum* day 4 tachyzoites with *T. gondii* day 4 tachyzoites) biological replicates were used for both A and B to gain more accurate estimates of experimental variation. Genes with an adjusted p-value of <1e-5 were considered differentially expressed. When considering differential expression between species rather than between different time points in the same species we considered only genes identified as pairwise orthologues. Orthologues of *N. caninum* and *T. gondii* are often different lengths and therefore we normalised the read counts for *T. gondii* genes based on the gene length of the *N. caninum* orthologue.

### Divergence time between species

We determined orthologous relationships between *N. caninum*, *T. gondii*, *P. falciparum* and *P. reichenowi* using orthoMCL [69]. Orthologous groups containing a single gene from each species were aligned using muscle [79] and those with less than 50% conserved positions across all four species (including gaps) were excluded, leaving 184 alignments. We further excluded those orthologous groups which we determined have not evolved in a clock-like manner. To do this we used a likelihood ratio test for a constant rate of evolution [80]. Likelihood computations on a fixed species tree under a model where branch lengths are free to vary, and under a model in which branch lengths were constrained to be clock-like were performed in PAUP 4b10 [81], under a general time-reversible model [82] incorporating both a proportion of invariant sites and a gamma distribution of rates across sites [83], [84]. The GTR+I+G model of evolution was applied for each locus independently. We performed this test for single-copy orthologous sequences, determined as above, from *T. gondii*, *N. caninum*, *P. falciparum*, *P. reichenowi* and *P. berghei*. Twelve of the 184 alignments were excluded because they were determined to have evolved in a nonclock-like manner. As clock-like evolution is the null hypothesis in this test, failure to reject a molecular clock can be either due to the true process of evolution being clock-like, or very close to clock-like for a particular locus, or because of a lack of power in the data to reject the clock. To test that this was not introducing a significant bias in our results, we looked at the estimate of divergence times from two different sets of loci: all loci that fail to reject the clock model, and only those genes in the top quartile of alignment length (which should have the most statistical power to reject the clock).

Codeml [85] was used to calculate the maximum likelihood value of dS in pairwise runmode with the JTT model allowing 2 or more dN/dS ratios for branches. Using all 172 alignments the median 4-fold coding site synonymous substitution rate (dS) across pairs of *N. caninum*/*T. gondii* orthologues was 0.856 substitutions per site. Between *P. falciparum* and *P. reichenowi* this was 0.076, similar to that calculated by Neafsey et al. [86] (0.068; 95% CI [0.060–0.077]). We assume that these Plasmodia diverged 2.49 mya (95% CI [1.93–3.79]) [30]. We thus dated the split for *N. caninum* and *T. gondii* to 28 mya, or taking into account the confidence intervals for the *Plasmodium* divergence estimate, between 21.7 and 42.7 mya, after the divergence of the definitive hosts around 52.9 mya [87].

If we calculate the median dS values using only those longest 25% of the 172 orthologous groups, we get a dS of 1.230 for *N. caninum*/*T. gondii* and 0.114 for *P. falciparum*/*P. reichenowi*. This translates to a divergence time of 26.9 mya, This value is very close to that calculated using all 172 alignments. This suggests that a any tendency to exclude longer genes using the clock test has not biased our results.

### Comparison of metabolic capacity

Enzyme Commission (EC) number mappings were extracted from the KEGG database [88] from 23 different species covering prokaryotes, archaea and eukaryotes and were mapped on to the corresponding genes in OrthoMCL database [69]. All *N. caninum* proteins that shared orthology with these enzymes were transitively assigned one or more EC number.

KEGG pathway mapping/coloring tools were used to map EC numbers to pathways. The final set of *N. caninum* metabolic pathways was compared to that of *T. gondii* (EC numbers assigned and used in similar fashion to *Neospora*). Pathways containing significantly high numbers of gene expression differences were determined as discussed in *Statistical analysis*.

### IRG phosphorylation assay

Cell culture was performed as described in [37]. The following immunoreagents were used (dilutions in parentheses). From J.C. Howard (University of Cologne): mouse anti-Irga6 monoclonal antibody (mAb) 10E7 (1∶500) [89], anti Irga6 phosphopeptide Ab T102-555 (1∶5000) [23] Alexa 350/488/546/555/647-labelled donkey anti-mouse, rabbit and goat sera (Molecular Probes), donkey anti-rabbit- (GE Healthcare), donkey antigoat- (Santa Cruz Biotechnology) and goat anti-mouse- HRP (horseradish peroxidase) (Pierce) antisera (all 1∶1000). From P. Bradley (UCLA): mouse anti-*N. caninum* ROP2 family member monoclonal antibody 20B5D5 (1∶2000). 4′,6-Diamidine-2′-phenylindole dihydrochloride (DAPI, Invitrogen) was used for nuclear counterstaining at a final concentration of 0.5 mg ml^−1^. Saponin permeabilization and immunostaining were performed as described in [90], [91], except for slides stained with T102-555 which were permeablized in ice cold methanol for 20 min and blocked in 1% BSA in PBS for 30 min.

### 1D SDS-PAGE

*N. caninum* proteins were purified from a tachyzoite pellet and resolved into 127 contiguous bands using acrylamide gel electrophoresis. Bands were excised and digested with trypsin. LC MS/MS was carried out using an LTQ ion trap mass spectrometer (Thermo Fisher Scientific Inc, Waltham, MA, USA) with an electrospray ionization source, Tryptic peptides were eluted using a linear gradient of 0–50% (v/v) acetonitrile/0.1% (v/v) formic acid over 140 minutes followed by 100% (v/v) ACN/0.1% formic acid for 20 minutes and a further 20 minutes of 0% (v/v) acetonitrile/0.1% (v/v) formic acid. Protein identifications were made as in [92], those above 1% false discovery rate were discarded. 1053 proteins were found to have at least one significantly matching peptide.

### Statistical analysis

To determine whether certain gene functions were overrepresented in differentially expressed genes we assigned GO terms using InterPro2GO [93]. The hypergeometric test was used to determine overrepresented GO terms in pooled day three and four expression data with a DESeq q-value cutoff of 1e^−5^. The Benjamini-Hochberg method was used to correct for multiple hypothesis testing. Values of P<0.05 were considered significant. The hypergeometric test was also used in the same way to determine whether KEGG metabolic pathways were enriched in differentially expressed genes.

## Supporting Information

Figure S1 **Alignment of Toxoplasma-Specfic Family (TSF).** Only sequences belonging to the Me49 strain of *T. gondii* are shown. Alignments here and in Supplementary Figure 2 were performed using Muscle [1] and displayed using Jalview [2]. Colouring is in Clustal format. TgTSF1 - TGME49_121170, TgTSF2 -TGME49_000700, TgTSF3 - TGME49_107260, TgTSF4 - TGME49_000130, TgTSF5 - TGME49_098960, TgTSF6 - TGME49_000590, TgTSF7 - TGME49_092710, TgTSF8 - TGME49_020080, TgTSF9 - TGME49_092810, TgTSF10 - TGME49_028780.
(EPS)

Figure S2 **Alignment of members of Lysine-Arginine rich Unidentified Function (KRUF).** See Figure S legend for details. TgKRUF1 - TGME49_092400, TgKRUF2 - TGME49_010590, TgKRUF3 - TGME49_092390, TgKRUF4 - TGME49_051170, TgKRUF5 - TGME49_051060, TgKRUF6 - TGME49_095940, TgKRUF7 - TGME49_052180, NcKRUF8 - NCLIV_002020, NcKRUF9 - NCLIV_043740, NcKRUF10 - NCLIV_002030.
(EPS)

Figure S3 **Distribution of SRS domain architecture subfamilies.** Alignments of each chromosome are shown with *N. caninum* above *T. gondii*. Pseudogenes are not shown in this figure. ‘VEG’ identifies loci where genes differ between Me49 and VEG strains of *T. gondii*. N.b. Note an expansion of family 3 in T. gondii Me49 at the start of chromosome VI. Family 3 has only four cysteines, rather than the six normally found in SAG domains (Wasmuth et al., submitted).
(EPS)

Figure S4 **Evidence of gene conversion at the SRS19 locus but not at the SRS29 locus.** Genes at the SRS19 locus cluster based on species rather than as orthologous pairs suggesting they have been subject to gene conversion (A). *N. caninum* genes are highlighted in purple. Genes from three representative strains of *T. gondii* are shown (ME49, GT1, VEG). The SRS37 locus is used here as an outgroup and does not show evidence of gene conversion. At the SRS29 locus genes have maintained independent lineages within each genome (B). Alignments were built using the PROMALS3D software with available SRS domain 3D structures. The phylogeny was inferred using Minimum Evolution and the evolutionary distances were calculated using the JTT matrix in MEGA4 [3]. The Greek letter psi indicates a pseudogene.
(EPS)

Figure S5 ***De novo*** **identification of non-coding RNAs.** The methodology used to identify ncRNAs is shown in A. B shows the overlap in those RNA candidates predicted to have structure by three different methods RNAz [4], Pfold [5] and QRNA [6]. Further details are given in the SOM text. HSP = High-scoring Segment Pair.
(EPS)

Figure S6 **Alignment and predicted structure of a novel ncRNA expanded in** ***N. caninum*** **.** Alignment of predicted novel ncRNA group 56 (A). The secondary structure was predicted by the program RNAalifold from the Vienna Package [7] and shown below as less than’ and ‘greater than’ symbols. Residues able to form base pairs according to the predicted structure are highlighted in black. Predicted structures are shown for both forward strand (left) and reverse strand (right) (B). Variable positions in stems are circled and the number of different base pairs (in the alignment) supporting a given structure is indicated by colors as described in [4].
(EPS)

Figure S7 **Identifying a threshold for calling gene expression.** We observed noisy mRNA-seq read mapping e.g. to intergenic and intronic sequence. We could not therefore rely on only a single read to call a gene as expressed. In order to determine a threshold expression level above which we could confidently call a gene as expressed we examined levels of expression in coding vs. non-coding regions of the *N. caninum* genome. This figure shows *N. caninum* chromosome VIII. 90% of intronic sequences were expressed with RPKM<6, while only 35% of exonic sequences had RPKM<6.
(EPS)

Figure S8 **Evidence for overlapping UTR sequences in convergently transcribed genes in both** ***N. caninum*** **and** ***T. gondii*** **.** We identified at least one convincing example of overlapping UTRs although we believe this is probably a rare occurrence in *T. gondii* and *N. caninum*. We show the example of NCLIV_009490 and NCLIV_009500 in *N. caninum* and their syntenic orthologues TGME49_098990 and TGME49_098980 in *T. gondii*. The putative product descriptions of these genes are ferredoxin NADP+ oxidoreductase and RNA pseudouridylate synthase. An asterisk highlights a region of continuous read depth between two gene models suggesting that their UTRs may overlap. This figure was created using RNAseq runs “Tg Day 3 TZ B” and “Nc Day 4 TZ A” remapped with TopHat [8] using parameters -r 250 -I 10000. The BLAST similarity was generated using TBLASTX with a score cutoff of 180, deeper reds relate to higher scores. ACT [9] was used to generate the figure.
(EPS)

Figure S9 **Overrepresented GO terms in genes differentially expressed between days three and six of** ***N. caninum*** **tachyzoite culture.** The term “DNA binding” largely refers to ApiAP2 transcription factor genes. “Protein amino acid phosphorylation” includes several rhoptry genes.
(EPS)

Table S1 **Species-specific genes with functional information and additional evidence.** RPKM values are means between replicates and are cut off below 6, as this was determined to be the minimum significant expression level. For *Neospora* we show RPKM values for days three, four and six of the tachyzoite stage and for *Toxoplasma* days three and four. *T. gondii* transcript abundances are from the VEG strain while *T. gondii* proteomics data are all those available in ToxoDb v6.4. *N. caninum* peptide data are from our own experiments.
(DOCX)

Table S2 **Frequencies of different SAG domain architectures.** SRS genes comprise one or more copies of the SAG domain. The SAG domain superfamily has been classified into eight subfamilies Fam1 to Fam8 (Wasmuth et al., submitted). The domain architectures of SRS genes and their frequency of occurrence in the *Neospora* and *Toxoplasma* genomes are described below. Each domain architecture is described in order from 5′ to 3′. Where many copies of a domain subfamily are present in succession, the number is indicated in brackets in the first column. Pseudogenes are excluded.
(DOCX)

Table S3 ***N. caninum*** **isolates for which the ROP18 region was amplified and sequenced.**
(DOCX)

Table S4 **Identity and orthologous relationships for apical complex genes and AP2 transcription factors.** *T. gondii* genes whose products are known to localize to apical complex organelles and their homologues (and AP2 transcription factors) are listed here. Where they exist, *N. caninum* othologues are identified and it is noted whether they occur in synteny or on another chromosome. We and others have identified some novel homologues in *N. caninum* and these are also included.
(DOCX)

Table S5 **RNAseq mapping statistics for** ***N. caninum*** **Liverpool tachyzoites.** TZ = tachyzoite. Sequencing was performed using an Illumina GAII as described in methods. Raw reads for these runs are available from the European Nucleotide Archive (ENA; http://www.ebi.ac.uk/ena/) under the run id listed. In library names A and B refer to biological replicates for a particular timepoint derived from distinct cultures of parasites.
(DOCX)

Table S6 **RNAseq mapping statistics for** ***T. gondii*** **VEG tachyzoites.** *T. gondii* RNAseq experiments were performed using the VEG strains due to their amenity in the lab, while the Me49 genome was used for mapping due to its superior assembly and annotation. Example day three and four RNAseq runs were mapped to the *T. gondii* VEG strain genome sequence to determine whether mapping was significantly improved relative to Me49. Mapping was very similar in both cases suggesting that mapping VEG transcriptome data to Me49 was a reasonable approach.
(DOCX)

Table S7 **Varieties of tRNA genes found in** ***T. gondii*** **Me40 and** ***N. caninum*** **Nc-Liv genomes.**
(DOCX)

Text S1 **Putative early bradyzoite differentiation in** ***N. caninum*** **at day six of culture.**
(DOC)

Text S2 **Identifying Non-coding RNA genes.**
(DOC)

Text S3 **References for Tables S1, S2, S3, S4, S5, S6.**
(DOCX)

## References

[1] Jones JL, Kruszon-Moran D, Wilson M, McQuillan G, Navin T (2001). Toxoplasma gondii infection in the United States: seroprevalence and risk factors.. Am J Epidemiol.

[2] Mandell GL, Douglas RGJ, Bennet JE (2010). Principles and practice of infectious diseases.

[3] Saeij JP, Coller S, Boyle JP, Jerome ME, White MW (2007). Toxoplasma co-opts host gene expression by injection of a polymorphic kinase homologue.. Nature.

[4] Taylor S, Barragan A, Su C, Fux B, Fentress SJ (2006). A secreted serine-threonine kinase determines virulence in the eukaryotic pathogen Toxoplasma gondii.. Science.

[5] Grigg ME, Bonnefoy S, Hehl AB, Suzuki Y, Boothroyd JC (2001). Success and virulence in Toxoplasma as the result of sexual recombination between two distinct ancestries.. Science.

[6] Su C, Evans D, Cole RH, Kissinger JC, Ajioka JW (2003). Recent expansion of Toxoplasma through enhanced oral transmission.. Science.

[7] Dubey JP, Carpenter JL, Speer CA, Topper MJ, Uggla A (1988). Newly recognized fatal protozoan disease of dogs.. J Am Vet Med Assoc.

[8] Dubey JP, Barr BC, Barta JR, Bjerkas I, Bjorkman C (2002). Redescription of Neospora caninum and its differentiation from related coccidia.. Int J Parasitol.

[9] McAllister MM, Dubey JP, Lindsay DS, Jolley WR, Wills RA (1998). Dogs are definitive hosts of Neospora caninum.. Int J Parasitol.

[10] McCann CM, Vyse AJ, Salmon RL, Thomas D, Williams DJ (2008). Lack of serologic evidence of Neospora caninum in humans, England.. Emerg Infect Dis.

[11] Dubey JP, Schares G, Ortega-Mora LM (2007). Epidemiology and control of neosporosis and Neospora caninum.. Clin Microbiol Rev.

[12] Davison HC, Otter A, Trees AJ (1999). Estimation of vertical and horizontal transmission parameters of Neospora caninum infections in dairy cattle.. Int J Parasitol.

[13] Trees AJ, Davison HC, Innes EA, Wastling JM (1999). Towards evaluating the economic impact of bovine neosporosis.. Int J Parasitol.

[14] Carruthers VB, Sibley LD (1997). Sequential protein secretion from three distinct organelles of Toxoplasma gondii accompanies invasion of human fibroblasts.. Eur J Cell Biol.

[15] Jung C, Lee CY, Grigg ME (2004). The SRS superfamily of Toxoplasma surface proteins.. Int J Parasitol.

[16] Keeley A, Soldati D (2004). The glideosome: a molecular machine powering motility and host-cell invasion by Apicomplexa.. Trends Cell Biol.

[17] Morisaki JH, Heuser JE, Sibley LD (1995). Invasion of Toxoplasma gondii occurs by active penetration of the host cell.. J Cell Sci.

[18] Dobrowolski JM, Sibley LD (1996). Toxoplasma invasion of mammalian cells is powered by the actin cytoskeleton of the parasite.. Cell.

[19] Alexander DL, Mital J, Ward GE, Bradley P, Boothroyd JC (2005). Identification of the moving junction complex of Toxoplasma gondii: a collaboration between distinct secretory organelles.. PLoS Pathog.

[20] Sinai AP, Joiner KA (2001). The Toxoplasma gondii protein ROP2 mediates host organelle association with the parasitophorous vacuole membrane.. J Cell Biol.

[21] Ong YC, Reese ML, Boothroyd JC (2010). Toxoplasma rhoptry protein 16 (ROP16) subverts host function by direct tyrosine phosphorylation of STAT6.. J Biol Chem.

[22] El Hajj H, Lebrun M, Arold ST, Vial H, Labesse G (2007). ROP18 is a rhoptry kinase controlling the intracellular proliferation of Toxoplasma gondii.. PLoS Pathog.

[23] Steinfeldt T, Konen-Waisman S, Tong L, Pawlowski N, Lamkemeyer T (2010). Phosphorylation of mouse immunity-related GTPase (IRG) resistance proteins is an evasion strategy for virulent Toxoplasma gondii.. PLoS Biol.

[24] Fentress SJ, Behnke MS, Dunay IR, Mashayekhi M, Rommereim LM (2010). Phosphorylation of immunity-related GTPases by a Toxoplasma gondii-secreted kinase promotes macrophage survival and virulence.. Cell Host Microbe.

[25] Gajria B, Bahl A, Brestelli J, Dommer J, Fischer S (2008). ToxoDB: an integrated Toxoplasma gondii database resource.. Nucleic Acids Res.

[26] DeBarry JD, Kissinger JC (2011). Jumbled genomes: missing Apicomplexan synteny.. Mol Biol Evol.

[27] Bahl A, Davis PH, Behnke M, Dzierszinski F, Jagalur M (2010). A novel multifunctional oligonucleotide microarray for Toxoplasma gondii.. BMC Genomics.

[28] Hunter S, Apweiler R, Attwood TK, Bairoch A, Bateman A (2009). InterPro: the integrative protein signature database.. Nucleic Acids Res.

[29] Ellis J, Luton K, Baverstock PR, Brindley PJ, Nimmo KA (1994). The phylogeny of Neospora caninum.. Mol Biochem Parasitol.

[30] Ricklefs RE, Outlaw DC (2010). A molecular clock for malaria parasites.. Science.

[31] Hedges SB, Dudley J, Kumar S (2006). TimeTree: a public knowledge-base of divergence times among organisms.. Bioinformatics.

[32] Kim SK, Boothroyd JC (2005). Stage-specific expression of surface antigens by Toxoplasma gondii as a mechanism to facilitate parasite persistence.. J Immunol.

[33] Boothroyd JC (2009). Expansion of host range as a driving force in the evolution of Toxoplasma.. Mem Inst Oswaldo Cruz.

[34] Howe DK, Crawford AC, Lindsay D, Sibley LD (1998). The p29 and p35 immunodominant antigens of Neospora caninum tachyzoites are homologous to the family of surface antigens of Toxoplasma gondii.. Infect Immun.

[35] Pollard AM, Onatolu KN, Hiller L, Haldar K, Knoll LJ (2008). Highly polymorphic family of glycosylphosphatidylinositol-anchored surface antigens with evidence of developmental regulation in Toxoplasma gondii.. Infect Immun.

[36] Khan A, Taylor S, Ajioka JW, Rosenthal BM, Sibley LD (2009). Selection at a single locus leads to widespread expansion of Toxoplasma gondii lineages that are virulent in mice.. PLoS Genet.

[37] Khaminets A, Hunn JP, Konen-Waisman S, Zhao YO, Preukschat D (2010). Coordinated loading of IRG resistance GTPases on to the Toxoplasma gondii parasitophorous vacuole.. Cell Microbiol.

[38] Butcher BA, Fox BA, Rommereim LM, Kim SG, Maurer KJ (2011). Toxoplasma gondii Rhoptry Kinase ROP16 Activates STAT3 and STAT6 Resulting in Cytokine Inhibition and Arginase-1-Dependent Growth Control.. PLoS Pathog.

[39] Yamamoto M, Standley DM, Takashima S, Saiga H, Okuyama M (2009). A single polymorphic amino acid on Toxoplasma gondii kinase ROP16 determines the direct and strain-specific activation of Stat3.. J Exp Med.

[40] Reese ML, Zeiner GM, Saeij JP, Boothroyd JC, Boyle JP (2011). Polymorphic family of injected pseudokinases is paramount in Toxoplasma virulence.. Proc Natl Acad Sci U S A.

[41] Dunn JD, Ravindran S, Kim SK, Boothroyd JC (2008). The Toxoplasma gondii dense granule protein GRA7 is phosphorylated upon invasion and forms an unexpected association with the rhoptry proteins ROP2 and ROP4.. Infect Immun.

[42] Shaw MK, Roos DS, Tilney LG (2002). Cysteine and serine protease inhibitors block intracellular development and disrupt the secretory pathway of Toxoplasma gondii.. Microbes Infect.

[43] Bradley PJ, Hsieh CL, Boothroyd JC (2002). Unprocessed Toxoplasma ROP1 is effectively targeted and secreted into the nascent parasitophorous vacuole.. Mol Biochem Parasitol.

[44] Naguleswaran A, Muller N, Hemphill A (2003). Neospora caninum and Toxoplasma gondii: a novel adhesion/invasion assay reveals distinct differences in tachyzoite-host cell interactions.. Exp Parasitol.

[45] Painter HJ, Campbell TL, Llinas M (2011). The Apicomplexan AP2 family: integral factors regulating Plasmodium development.. Mol Biochem Parasitol.

[46] Yuda M, Iwanaga S, Shigenobu S, Kato T, Kaneko I (2010). Transcription factor AP2-Sp and its target genes in malarial sporozoites.. Mol Microbiol.

[47] Altschul SF, Wootton JC, Zaslavsky E, Yu YK (2010). The construction and use of log-odds substitution scores for multiple sequence alignment.. PLoS Comput Biol.

[48] De Silva EK, Gehrke AR, Olszewski K, Leon I, Chahal JS (2008). Specific DNA-binding by apicomplexan AP2 transcription factors.. Proc Natl Acad Sci U S A.

[49] Behnke MS, Wootton JC, Lehmann MM, Radke JB, Lucas O (2010). Coordinated progression through two subtranscriptomes underlies the tachyzoite cycle of Toxoplasma gondii.. PLoS One.

[50] Wasmuth J, Daub J, Peregrin-Alvarez JM, Finney CA, Parkinson J (2009). The origins of apicomplexan sequence innovation.. Genome Res.

[51] Ewald PW (1993). Evolution of Infectious Disease.

[52] Stewart AD, Logsdon JM, Kelley SE (2005). An empirical study of the evolution of virulence under both horizontal and vertical transmission.. Evolution.

[53] Hunn JP (2007). Evolution and Cellular Resistance Mechanisms of the Immunity-Related GTPases [PhD thesis].

[54] Barber JS, Holmdahl OJ, Owen MR, Guy F, Uggla A (1995). Characterization of the first European isolate of Neospora caninum (Dubey, Carpenter, Speer, Topper and Uggla).. Parasitology.

[55] Cohen AM, Rumpel K, Coombs GH, Wastling JM (2002). Characterisation of global protein expression by two-dimensional electrophoresis and mass spectrometry: proteomics of Toxoplasma gondii.. Int J Parasitol.

[56] Batzoglou S, Jaffe DB, Stanley K, Butler J, Gnerre S (2002). ARACHNE: a whole-genome shotgun assembler.. Genome Res.

[57] Delcher AL, Phillippy A, Carlton J, Salzberg SL (2002). Fast algorithms for large-scale genome alignment and comparison.. Nucleic Acids Res.

[58] Kozarewa I, Ning Z, Quail MA, Sanders MJ, Berriman M (2009). Amplification-free Illumina sequencing-library preparation facilitates improved mapping and assembly of (G+C)-biased genomes.. Nat Methods.

[59] Ning Z, Cox AJ, Mullikin JC (2001). SSAHA: a fast search method for large DNA databases.. Genome Res.

[60] Korf I (2004). Gene finding in novel genomes.. BMC Bioinformatics.

[61] Stanke M, Schoffmann O, Morgenstern B, Waack S (2006). Gene prediction in eukaryotes with a generalized hidden Markov model that uses hints from external sources.. BMC Bioinformatics.

[62] Pertea M, Lin X, Salzberg SL (2001). GeneSplicer: a new computational method for splice site prediction.. Nucleic Acids Res.

[63] Majoros WH, Pertea M, Salzberg SL (2004). TigrScan and GlimmerHMM: two open source ab initio eukaryotic gene-finders.. Bioinformatics.

[64] Korf I, Flicek P, Duan D, Brent MR (2001). Integrating genomic homology into gene structure prediction.. Bioinformatics.

[65] Allen JE, Salzberg SL (2005). JIGSAW: integration of multiple sources of evidence for gene prediction.. Bioinformatics.

[66] Haas BJ, Salzberg SL, Zhu W, Pertea M, Allen JE (2008). Automated eukaryotic gene structure annotation using EVidenceModeler and the Program to Assemble Spliced Alignments.. Genome Biol.

[67] Quackenbush J, Cho J, Lee D, Liang F, Holt I (2001). The TIGR Gene Indices: analysis of gene transcript sequences in highly sampled eukaryotic species.. Nucleic Acids Res.

[68] Carver T, Berriman M, Tivey A, Patel C, Bohme U (2008). Artemis and ACT: viewing, annotating and comparing sequences stored in a relational database.. Bioinformatics.

[69] Li L, Stoeckert CJ, Roos DS (2003). OrthoMCL: identification of ortholog groups for eukaryotic genomes.. Genome Res.

[70] Bradley PJ, Ward C, Cheng SJ, Alexander DL, Coller S (2005). Proteomic analysis of rhoptry organelles reveals many novel constituents for host-parasite interactions in Toxoplasma gondii.. J Biol Chem.

[71] Boothroyd JC, Dubremetz JF (2008). Kiss and spit: the dual roles of Toxoplasma rhoptries.. Nat Rev Microbiol.

[72] Dowse T, Soldati D (2004). Host cell invasion by the apicomplexans: the significance of microneme protein proteolysis.. Curr Opin Microbiol.

[73] Friedrich N, Santos JM, Liu Y, Palma AS, Leon E (2010). Members of a novel protein family containing microneme adhesive repeat domains act as sialic acid-binding lectins during host cell invasion by apicomplexan parasites.. J Biol Chem.

[74] Sohn CS, Cheng TT, Drummond ML, Peng ED, Vermont SJ (2011). Identification of novel proteins in Neospora caninum using an organelle purification and monoclonal antibody approach.. PLoS One.

[75] Michelin A, Bittame A, Bordat Y, Travier L, Mercier C (2009). GRA12, a Toxoplasma dense granule protein associated with the intravacuolar membranous nanotubular network.. Int J Parasitol.

[76] Lebrun M, Carruthers VB, Cesbron-Delauw MF, Weiss LM, Kim K (2007). Toxoplasma Secretory Proteins and Their Roles in Cell Invasion..

[77] Altschul SF, Wootton JC, Zaslavsky E, Yu YK (2010). The construction and use of log-odds substitution scores for multiple sequence alignment.. PLoS Comput Biol.

[78] Anders S, Huber W (2010). Differential expression analysis for sequence count data.. Genome Biol.

[79] Edgar RC (2004). MUSCLE: multiple sequence alignment with high accuracy and high throughput.. Nucleic Acids Res.

[80] Felsenstein J (1981). Evolutionary trees from DNA sequences: a maximum likelihood approach.. J Mol Evol.

[81] Swofford DL (2002). PAUP*. Phylogenetic Analysis Using Parsimony (*and Other Methods).

[82] Tavare S, Miura RM (1986). Some probabilistic and statistical problems in the analysis of DNA sequences.. Some mathematical questions in biology - DNA sequence analysis.

[83] Yang Z (1994). Maximum likelihood phylogenetic estimation from DNA sequences with variable rates over sites: approximate methods.. J Mol Evol.

[84] Hasegawa M, Kishino H, Yano T (1985). Dating of the human-ape splitting by a molecular clock of mitochondrial DNA.. J Mol Evol.

[85] Yang Z (2007). PAML 4: phylogenetic analysis by maximum likelihood.. Mol Biol Evol.

[86] Neafsey DE, Hartl DL, Berriman M (2005). Evolution of noncoding and silent coding sites in the Plasmodium falciparum and Plasmodium reichenowi genomes.. Mol Biol Evol.

[87] Eizirik E, Murphy W, Hedges SB, Kumar S (2009). Carnivores (Carnivora).. The Timetree of Life.

[88] Kanehisa M, Goto S (2000). KEGG: kyoto encyclopedia of genes and genomes.. Nucleic Acids Res.

[89] Papic N, Hunn JP, Pawlowski N, Zerrahn J, Howard JC (2008). Inactive and active states of the interferon-inducible resistance GTPase, Irga6, in vivo.. J Biol Chem.

[90] Martens S, Parvanova I, Zerrahn J, Griffiths G, Schell G (2005). Disruption of Toxoplasma gondii parasitophorous vacuoles by the mouse p47-resistance GTPases.. PLoS Pathog.

[91] Martens S, Sabel K, Lange R, Uthaiah R, Wolf E (2004). Mechanisms regulating the positioning of mouse p47 resistance GTPases LRG-47 and IIGP1 on cellular membranes: retargeting to plasma membrane induced by phagocytosis.. J Immunol.

[92] Jones AR, Siepen JA, Hubbard SJ, Paton NW (2009). Improving sensitivity in proteome studies by analysis of false discovery rates for multiple search engines.. Proteomics.

[93] Camon EB, Barrell DG, Dimmer EC, Lee V, Magrane M (2005). An evaluation of GO annotation retrieval for BioCreAtIvE and GOA.. BMC Bioinformatics.

[94] Hertz-Fowler C, Peacock CS, Wood V, Aslett M, Kerhornou A (2004). GeneDB: a resource for prokaryotic and eukaryotic organisms.. Nucleic Acids Res.

