# Supplementary material for: Comparative Genomics of the Apicomplexan Parasites Toxoplasma gondii and Neospora caninum: Coccidia Differing in Host Range and Transmission Strategy
Source: PLoS Pathog. 2012 Mar 22;8(3):e1002567. doi: 10.1371/journal.ppat.1002567 (PMC3310773; doi:10.1371/journal.ppat.1002567)
Supplement: Table S1 — Species-specific genes with functional information and additional evidence. RPKM values are means between replicates and are cut off below 6, as this was determined to be the minimum significant expression level. For Neospora we show RPKM values for days three, four and six of the tachyzoite stage and for Toxoplasma days three and four. T. gondii transcript abundances are from the VEG strain while T. gondii proteomics data are all those available in ToxoDb v6.4. N. caninum peptide data are from our own experiments. (DOCX) [file ppat.1002567.s010.docx]

Supplementary Table 1.Species-specific genes with functional information and additional evidence.

RPKM values are means between replicates and are cut off below 6, as this was determined to be the minimum significant expression level. For *Neospora* we show RPKM values for days three, four and six of the tachyzoite stage and for *Toxoplasma* days three and four. *T. gondii* transcript abundances are from the VEG strain while *T. gondii* proteomics data are all those available in ToxoDb v6.4. *N. caninum* peptide data are from our own experiments.

| **Gene Name** | **Gene Id** | **Description** | **Peptide hits** | **Gene expression (RPKM)** | **Pfam domains** |
| --- | --- | --- | --- | --- | --- |
|  |  |  |  |  |  |
| ***N. caninum*** |  |  |  |  |  |
|  |  |  |  |  |  |
| ROP1B | NCLIV_069110 | ROP1 homologue | 43 | 18/0/0 | - |
| ROP5B | NCLIV_060740 | ROP2 homologue | 0 | 1785/1513/472 | Protein kinase domain |
| ROP15B | NCLIV_011700 | ROP2 homologue | 37 | 524/287/75 | Protein kinase domain |
| ROP51 | NCLIV_068890 | ROP2 homologue | 22 | 560/209/11 | Protein kinase domain |
| ROP52 | NCLIV_069590 | ROP2 homologue | 30 | 720/166/10 | Protein kinase domain |
| ROP53 | NCLIV_068850 | ROP2 homologue | 17 | 349/155/16 | Protein kinase domain |
| ROP55 | NCLIV_031550 | ROP2 homologue, pseudogenised in *T. gondii* ME49 | 199 | 870/210/28 | Protein kinase domain |
| MIC19 | NCLIV_038320 | MIC4 homologue | 13 | 212/66/0 | PAN domain |
| MIC26 | NCLIV_033690 | Paralogue of NcMIC2 | 63 | 2940/3057/1924 | Thrombospondin type-1 (TSP1) |
| MCP7 | NCLIV_054425 | MAR domain-containing protein [[10](#_ENREF_10)] | 0 | 21/8/9 | MAR domain-containing protein |
| MCP6 | NCLIV_054450 | MAR domain-containing protein [[10](#_ENREF_10)] | 0 | 0/0/0 | MAR domain-containing protein |
| MCP5 | NCLIV_066750 | MAR domain-containing protein [[10](#_ENREF_10)] | 0 | 0/0/0 | MAR domain-containing protein |
|  | NCLIV_042240 | hypothetical protein | 0 | 6/7/0 | Amino_oxidase |
|  | NCLIV_013200 | hypothetical protein | 0 | 2658/1108/2672 | ATP11 |
|  | NCLIV_060780 | hypothetical protein | 0 | 0/0/7 | CKS |
|  | NCLIV_002890 | hypothetical protein | 0 | 0/0/0 | DAO |
|  | NCLIV_069030 | hypothetical protein | 0 | 16/16/12 | DEAD |
|  | NCLIV_026620 | hypothetical protein | 0 | 0/0/8 | DHH |
|  | NCLIV_043010 | hypothetical protein | 0 | 0/0/0 | Dynein_light |
|  | NCLIV_048350 | ER lumen protein retaining receptor, related | 0 | 51/66/49 | ER_lumen_recept |
|  | NCLIV_064730 | hypothetical protein | 0 | 14/7/0 | EXS |
|  | NCLIV_027890 | hypothetical protein | 0 | 6/10/0 | FKBP_C |
|  | NCLIV_068740 | Glucose-6-phosphate 1-dehydrogenase (EC 1.1.1.49), related | 0 | 0/0/0 | G6PD_N |
|  | NCLIV_043670 | High mobility group protein, related | 3 | 758/557/155 | HMG_box |
|  | NCLIV_060790 | High mobility group protein 1, related | 0 | 6/12/9 | HMG_box |
|  | NCLIV_001720 | Lipocalin-like protein, related | 0 | 0/0/0 | Lipocalin_2 |
|  | NCLIV_029500 | hypothetical protein | 0 | 7/0/0 | Lipoprotein_7 |
|  | NCLIV_014880 | hypothetical protein | 0 | 0/0/0 | LRR_1 |
|  | NCLIV_035170 | hypothetical protein | 0 | 27/34/11 | MACPF |
|  | NCLIV_059810 | hypothetical protein | 0 | 31/30/55 | Nop25 |
|  | NCLIV_000970 | hypothetical protein | 0 | 0/0/0 | PDEase_I |
|  | NCLIV_037220 | hypothetical protein | 0 | 28/22/34 | Peptidase_C48 |
|  | NCLIV_068300 | GD23551, related | 0 | 11/0/0 | PIG-U |
|  | NCLIV_013080 | hypothetical protein | 0 | 0/0/0 | Pkinase |
|  | NCLIV_029900 | AGC family protein kinase, related | 0 | 0/0/0 | Pkinase |
|  | NCLIV_036570 | YALI0D21604p, related | 2 | 206/132/61 | PS_Dcarbxylase |
|  | NCLIV_068720 | 50S ribosomal protein L2, chloroplastic, related | 0 | 0/0/0 | Ribosomal_L2_C |
|  | NCLIV_069470 | hypothetical protein | 0 | 269/635/564 | Ribosomal_L41 |
|  | NCLIV_043800 | hypothetical protein | 0 | 21/25/35 | RNA_pol_Rpb7_N,RNA_pol_Rbc25 |
|  | NCLIV_009100 | hypothetical protein | 0 | 7/7/10 | RRM_1 |
|  | NCLIV_024340 | hypothetical protein | 0 | 12/30/64 | RRM_1 |
|  | NCLIV_047560 | hypothetical protein | 0 | 15/19/32 | S1 |
|  | NCLIV_058390 | hypothetical protein | 0 | 0/0/0 | SCAMP |
|  | NCLIV_013190 | hypothetical protein | 0 | 624/917/841 | Sec1 |
|  | NCLIV_047590 | hypothetical protein | 0 | 7/0/0 | Snf7 |
|  | NCLIV_022020 | Zinc finger (CCCH type) protein, related | 0 | 110/89/132 | zf-CCCH |
|  | NCLIV_050190 | Zinc finger (CCCH type) protein, related | 0 | 0/0/0 | zf-CCCH |
|  | NCLIV_061900 | Poly-zinc finger protein 1, related | 0 | 45/57/69 | zf-CCHC |
|  | NCLIV_068960 | hypothetical protein | 0 | 44/35/36 | zf-Sec23_Sec24,Sec23_trunk |
|  | NCLIV_043740 | hypothetical protein | 1 | 0/0/0 | - |
|  | NCLIV_046290 | hypothetical protein | 1 | 84/114/164 | - |
|  | NCLIV_069460 | hypothetical protein | 1 | 191/189/213 | - |
|  |  |  |  |  |  |
| ***T. gondii* Me49** |  |  |  |  |  |
|  |  |  |  |  |  |
| ROP2A | TGME49_015780 | Rhoptry kinase family protein ROP2A | 39 | 26898/10379 | - |
| ROP2B | TGME49_075300 | Rhoptry kinase family protein ROP2B | 27 | 6755/2675 | - |
| ROP8 | TGME49_015770 | Rhoptry kinase family protein ROP8 | 24 | 88/66 | - |
| ROP18 | TGME49_005250 | Resequencing of multiple N. caninum strains confirmed pseudogenisation | 50 | 0/0 | Protein kinase domain |
| ROP42 | TGME49_009980 | Rhoptry kinase family | 27 | 35/52 | - |
| ROP43 | TGME49_010090 | Rhoptry kinase family | 17 | 57/121 | - |
| BRP1 | TGME49_114250 | Pseudogenised in *N. caninum* | 0 | 8/9 | - |
| GRA11 | TGME49_012410 | Dense granule protein | 0 | 18/44 | - |
| GRA12 | TGME49_075850 | Dense granule protein | 0 | 0/8 | - |
| - | TGME49_104480 | 3-oxo-5 alpha-steroid delta 4-dehydrogenase alpha 2, putative | 0 | 0/6 | 3-oxo-5-alpha-steroid 4-dehydrogenase |
| - | TGME49_105800 | hypothetical protein, conserved | 0 | 16/15 | 6-pyruvoyl tetrahydropterin synthase |
| - | TGME49_056790 | hypothetical protein | 0 | 0/0 | ABC-type uncharacterized transport system |
| - | TGME49_034510 | acyl-CoA-binding protein, putative | 4 | 82/108 | Acyl CoA binding protein,Ankyrin repeat |
| - | TGME49_103450 | hypothetical protein, conserved | 0 | 0/0 | Adaptin N terminal region |
| - | TGME49_117600 | ankyrin repeat-containing protein | 0 | 0/0 | Ankyrin repeat |
| - | TGME49_024180 | ankyrin repeat-containing protein | 0 | 0/0 | Ankyrin repeat,Ankyrin repeat,BTB/POZ domain |
| - | TGME49_023250 | vacuolar type H+-ATPase proteolipid subunit, putative | 0 | 0/0 | ATP synthase subunit C,ATP synthase subunit C |
| - | TGME49_102150 | ATPase, AAA family domain-containing protein | 0 | 0/0 | ATPase family associated with various cellular activities (AAA) |
| - | TGME49_102000 | chaperone clpB protein, putative | 2 | 0/0 | ATPase family associated with various cellular activities (AAA),C-terminal, D2-small domain, of ClpB protein |
| - | TGME49_117000 | acetylcholinesterase, putative | 0 | 0/0 | Carboxylesterase |
| - | TGME49_103150 | CCR4-Not complex component, Not1 domain-containing protein | 0 | 0/0 | CCR4-Not complex component, Not1 |
| - | TGME49_067710 | hypothetical protein | 7 | 15/16 | CPSF A subunit region |
| - | TGME49_085460 | hypothetical protein, conserved | 0 | 0/0 | Cullin protein neddylation domain |
| - | TGME49_037130 | cytochrome b, putative | 2 | 106/78 | Cytochrome b(N-terminal)/b6/petB |
| - | TGME49_101370 | zinc finger DHHC domain-containing protein, conserved | 0 | 0/0 | DHHC zinc finger domain |
| - | TGME49_117200 | Fz domain-containing protein | 0 | 0/0 | Fz domain |
| - | TGME49_098610 | grb10 interacting GYF protein, putative | 33 | 75/99 | GYF domain |
| - | TGME49_102450 | TFIIH basal transcription factor complex helicase XPB subunit, putative | 0 | 0/0 | Helicase conserved C-terminal domain |
| - | TGME49_104350 | helicase conserved C-terminal domain-containing protein | 0 | 0/0 | Helicase conserved C-terminal domain,Helicase associated domain (HA2) |
| - | TGME49_004080 | histidine acid phosphatase domain containing protein | 4 | 92/100 | Histidine acid phosphatase |
| - | TGME49_067720 | hypothetical protein | 0 | 118/136 | Ion transport protein |
| - | TGME49_098600 | leucine zipper-like transcriptional regulator | 0 | 39/44 | Kelch motif,BTB/POZ domain |
| - | TGME49_105090 | hypothetical protein | 0 | 65/125 | Kinase binding protein CGI-121 |
| - | TGME49_018460 | hypothetical protein | 0 | 0/0 | Lectin C-type domain |
| - | TGME49_102550 | NUC173 domain-containing protein | 0 | 0/0 | NUC173 domain |
| - | TGME49_102750 | OB-fold nucleic acid binding domain-containing protein | 0 | 0/0 | OB-fold nucleic acid binding domain,tRNA synthetases class II (D, K and N) |
| - | TGME49_102650 | peptidase family M13 domain-containing protein | 0 | 0/0 | Peptidase family M13,Peptidase family M13 |
| - | TGME49_103350 | putative GTP-ase activating protein for Arf domain-containing protein | 0 | 0/0 | PH domain,Putative GTPase activating protein for Arf |
| - | TGME49_113850 | hypothetical protein | 0 | 0/0 | Protamine P1 |
| - | TGME49_062050 | Rhoptry kinase family protein ROP39 | 22 | 435/150 | Protein kinase domain |
| - | TGME49_104150 | protein kinase, putative | 0 | 0/0 | Protein kinase domain |
| - | TGME49_103550 | hypothetical protein | 0 | 0/0 | Putative GTPase activating protein for Arf |
| - | TGME49_096850 | putative tRNA binding domain-containing protein | 0 | 0/0 | Putative tRNA binding domain |
| - | TGME49_006360 | peptidyl-tRNA hydrolase domain-containing protein | 0 | 14/18 | RF-1 domain |
| - | TGME49_102055 | ribosomal protein S23, putative | 1 | 0/17 | Ribosomal protein S12 |
| - | TGME49_086610 | 30S ribosomal protein S14, putative | 0 | 10/12 | Ribosomal protein S14p/S29e |
| - | TGME49_100680 | RNA polymerase beta' chain | 0 | 0/0 | RNA polymerase Rpb1, domain 2 |
| - | TGME49_100690 | RNA polymerase Rpb1, domain 5 family protein | 0 | 0/0 | RNA polymerase Rpb1, domain 5, |
| - | TGME49_100660 | DNA dependent RNA polymerase beta subunit | 0 | 0/0 | RNA polymerase Rpb2, domain 3,RNA polymerase Rpb2, domain 6 |
| - | TGME49_100670 | hypothetical protein | 0 | 0/0 | RNA polymerase Rpb2, domain 6 |
| - | TGME49_088230 | hypothetical protein | 0 | 13/19 | Serine-threonine protein kinase 19 |
| - | TGME49_115350 | hypothetical protein, conserved | 0 | 0/0 | Snf7 |
| - | TGME49_098630 | SPX domain-containing protein | 34 | 57/75 | SPX domain,VTC domain,Domain of unknown function DUF |
| - | TGME49_095080 | hypothetical protein | 0 | 17/11 | TLC domain |
| - | TGME49_055720 | hypothetical protein | 0 | 0/7 | tRNA intron endonuclease, catalytic C-terminal domain |
| - | TGME49_003460 | hypothetical protein, conserved | 0 | 14/13 | Uncharacterised protein family (UPF0203) |
| - | TGME49_087200 | hypothetical protein | 0 | 18/17 | Uncharacterised protein family UPF0546 |
| - | TGME49_100650 | sufB/sufD domain-containing protein | 0 | 0/0 | Uncharacterized protein family (UPF0051) |
| - | TGME49_098460 | product unspecified | 0 | 0/0 | WD domain, G-beta repeat, |
| - | TGME49_088900 | Yos1-like domain-containing protein | 0 | 19/15 | Yos1-like |
| - | TGME49_103050 | Zinc finger, C2H2 type family protein | 0 | 0/0 | Zinc finger, C2H2 type, |
| - | TGME49_004340 | hypothetical protein | 29 | 365/459 | - |
| - | TGME49_104950 | hypothetical protein | 17 | 307/432 | - |
| - | TGME49_104670 | internalin, putative | 15 | 49/32 | - |
| - | TGME49_031960 | ppg3, putative | 14 | 79/44 | - |
| - | TGME49_052630 | hypothetical protein | 11 | 134/105 | - |
| - | TGME49_044170 | hypothetical protein | 7 | 25/37 | - |
| - | TGME49_030180 | hypothetical protein | 5 | 694/987 | - |
| - | TGME49_037880 | hypothetical protein | 4 | 407/92 | - |
| - | TGME49_067750 | hypothetical protein | 4 | 17/11 | - |
| - | TGME49_086510 | hypothetical protein | 4 | 17/12 | - |
| - | TGME49_116700 | hypothetical protein | 4 | 9/6 | - |
| - | TGME49_010810 | hypothetical protein | 3 | 81/121 | - |
| - | TGME49_029220 | hypothetical protein, conserved | 3 | 111/110 | - |
| - | TGME49_052480 | hypothetical protein | 3 | 11/19 | - |
| - | TGME49_075470 | hypothetical protein | 3 | 107/82 | - |
| - | TGME49_118370 | hypothetical protein | 3 | 23/24 | - |
| - | TGME49_013050 | hypothetical protein | 2 | 465/562 | - |
| - | TGME49_075990 | hypothetical protein | 2 | 53/78 | - |
| - | TGME49_113210 | hypothetical protein | 2 | 0/0 | - |
