# Supplementary material for: Comparative Genomics of the Apicomplexan Parasites Toxoplasma gondii and Neospora caninum: Coccidia Differing in Host Range and Transmission Strategy
Source: PLoS Pathog. 2012 Mar 22;8(3):e1002567. doi: 10.1371/journal.ppat.1002567 (PMC3310773; doi:10.1371/journal.ppat.1002567)
Supplement: Table S2 — Frequencies of different SAG domain architectures. SRS genes comprise one or more copies of the SAG domain. The SAG domain superfamily has been classified into eight subfamilies Fam1 to Fam8 (Wasmuth et al., submitted). The domain architectures of SRS genes and their frequency of occurrence in the Neospora and Toxoplasma genomes are described below. Each domain architecture is described in order from 5′ to 3′. Where many copies of a domain subfamily are present in succession, the number is indicated in brackets in the first column. Pseudogenes are excluded. (DOCX) [file ppat.1002567.s011.docx]

Supplementary Table 2. Frequencies of different SAG domain architectures

SRS genes comprise one or more copies of the SAG domain. The SAG domain superfamily has been classified into eight subfamilies Fam1 to Fam8 (Wasmuth et al., submitted). The domain architectures of SRS genes and their frequency of occurrence in the *Neospora* and *Toxoplasma* genomes are described below. Each domain architecture is described in order from 5´ to 3´. Where many copies of a domain subfamily are present in succession, the number is indicated in brackets in the first column. Pseudogenes are excluded.

|  | *N. caninum* Liverpool | *T. gondii* ME49 |
| --- | --- | --- |
| Fam7-Fam8 | 131 | 33 |
| Fam6-Fam2 | 29 | 7 |
| Fam2-Fam2 | 11 | 3 |
| Fam5-Fam1 | 8 | 6 |
| Fam6-Fam1 | 7 | 4 |
| Fam4-Fam2 | 7 | 3 |
| Fam2-Fam1 | 5 | 1 |
| Fam1-Fam2 | 5 | 0 |
| Fam8-Fam8 | 4 | 4 |
| Fam2 | 3 | 8 |
| Fam1 | 3 | 4 |
| Fam8 | 2 | 4 |
| Fam6 | 2 | 4 |
| Fam8-Fam7-Fam8 | 1 | 0 |
| Fam7-Fam8-Fam7-Fam8 | 1 | 0 |
| Fam7 | 1 | 0 |
| Fam6-Fam2-Fam2-Fam3-Fam2 | 1 | 0 |
| Fam5 | 1 | 4 |
| Fam4-Fam4-Fam4-Fam4-Fam2 | 1 | 0 |
| Fam4-Fam4-Fam2 | 1 | 5 |
| Fam2(11)-Fam3 | 1 | 0 |
| Fam2-Fam2-Fam2 | 1 | 0 |
| Fam3 | 1 | 10 |
| Fam6-Fam6-Fam2-Fam3-Fam2 | 0 | 1 |
| Fam4-Fam6-Fam2-Fam4-Fam2 | 0 | 1 |
| Fam2-Fam4-Fam2 | 0 | 1 |
| Fam2(13)-Fam3 | 0 | 1 |
| Totals | 227 | 104 |
