# Supplementary material for: Comparative Genomics of the Apicomplexan Parasites Toxoplasma gondii and Neospora caninum: Coccidia Differing in Host Range and Transmission Strategy
Source: PLoS Pathog. 2012 Mar 22;8(3):e1002567. doi: 10.1371/journal.ppat.1002567 (PMC3310773; doi:10.1371/journal.ppat.1002567)
Supplement: Table S3 — N. caninum isolates for which the ROP18 region was amplified and sequenced. (DOCX) [file ppat.1002567.s012.docx]

Supplementary Table 3. *N. caninum* isolates for which the ROP18 region was amplified and sequenced

| **Isolate** | **Country of origin** | **Tissue isolated from** | **Reference** |
| --- | --- | --- | --- |
| Nc Liverpool | UK | Canine | Barber et al., 1993 [[11](#_ENREF_11)] |
| Nc 1 | USA | Canine | Dubey et al., 1988 [[12](#_ENREF_12)] |
| Nc Liverpool B1 | UK | Bovine | Davidson et al., 1997 [[13](#_ENREF_13)] |
| Nc BPA | USA | Bovine | Conrad et al., 1993 [[14](#_ENREF_14)] |
| Nc JPA | Japan | Bovine | Yamane et al., 1996 [[15](#_ENREF_15)] |
