# Supplementary material for: Comparative Genomics of the Apicomplexan Parasites Toxoplasma gondii and Neospora caninum: Coccidia Differing in Host Range and Transmission Strategy
Source: PLoS Pathog. 2012 Mar 22;8(3):e1002567. doi: 10.1371/journal.ppat.1002567 (PMC3310773; doi:10.1371/journal.ppat.1002567)
Supplement: Table S4 — Identity and orthologous relationships for apical complex genes and AP2 transcription factors. T. gondii genes whose products are known to localize to apical complex organelles and their homologues (and AP2 transcription factors) are listed here. Where they exist, N. caninum othologues are identified and it is noted whether they occur in synteny or on another chromosome. We and others have identified some novel homologues in N. caninum and these are also included. (DOCX) [file ppat.1002567.s013.docx]

Supplementary Table 4. Identity and orthologous relationships for apical complex genes and AP2 transcription factors.

*T. gondii* genes whose products are known to localise to apical complex organelles and their homologues (and AP2 transcription factors) are listed here. Where they exist, *N. caninum* othologues are identified and it is noted whether they occur in synteny or on another chromosome. We and others have identified some novel homologues in *N. caninum* and these are also included.

| **Name** | **ToxoDb Id v5.2** | ***T. gondii* chromosome** | ***N. caninum* Id** | ***N. caninum* chromosome** |
| --- | --- | --- | --- | --- |
|  |  |  |  |  |
|  |  |  |  |  |
| **Rhoptry genes** | |  |  |  |
|  |  |  |  |  |
| BRP1 | TGME49_114250 | XI | - | - |
| NHE2 | TGME49_099060 | III | NCLIV_009440 | Syntenic |
| PP2C-hn | TGME49_082060 | VIIa | NCLIV_023710 | Syntenic |
| RAB11 | TGME49_089680 | IX | NCLIV_041930 | Syntenic |
| RON1 | TGME49_110010 | XI | NCLIV_054120 | Syntenic |
| RON2 | TGME49_100100 | XII | NCLIV_064620 | Syntenic |
| RON2L1 | TGME49_094400 | Ia | NCLIV_001400 | Syntenic |
| RON2L2 | TGME49_065120 | IX | NCLIV_040110 | Syntenic |
| RON3 | TGME49_023920 | X | NCLIV_048590 | Syntenic |
| RON3L1 | TGME49_005360 | VIIa | NCLIV_020330; NCLIV_020340 | Syntenic |
| RON4 | TGME49_029010 | VIII | NCLIV_030050 | Syntenic |
| RON4L1 | TGME49_053370 | III | NCLIV_007800 | Syntenic |
| RON5 | TGME49_111470 | XI | NCLIV_055360 | Syntenic |
| RON6 | TGME49_097960 | II | NCLIV_006850 | Syntenic |
| ROP1 | TGME49_109590 | XI | NCLIV_053840 | Syntenic |
| ROP1B | - | - | NCLIV_069110 | Unk |
| ROP2A | TGME49_015780 | X | - | - |
| ROP2B | TGME49_075300 | X | - | - |
| ROP4 | TGME49_095110 | Ia | NCLIV_001950 | Syntenic |
| ROP5 | TGME49_108080 | XII | NCLIV_060730 | Syntenic |
| ROP5B | - | - | NCLIV_060740 | XII |
| ROP6 | TGME49_058660 | VIIb | NCLIV_027850 | Syntenic |
| ROP7 | TGME49_095110 | Ia | NCLIV_001970 | Syntenic |
| ROP8 | TGME49_015770 | X | - | - |
| ROP9 | TGME49_043730 | VI | NCLIV_018420 | Syntenic |
| ROP10 | TGME49_115490 | XI | NCLIV_058180 | Syntenic |
| ROP11 | TGME49_027810 | X | NCLIV_045585 | Syntenic |
| ROP12 | TGME49_003990 | VIIa | NCLIV_021100 | Syntenic |
| ROP13 | TGME49_112270 | XI | NCLIV_055850 | Syntenic |
| ROP14 | TGME49_115220 | XI | NCLIV_057960 | Syntenic |
| ROP14B | TGME49_115210 | XI | NCLIV_057950 | Syntenic |
| ROP15 | TGME49_011290 | IV | NCLIV_011690 | Syntenic |
| ROP15B | - | - | NCLIV_011700 | IV |
| ROP16 | TGME49_062730 | VIIb | NCLIV_025120 | Syntenic |
| ROP17 | TGME49_058580 | VIIb | NCLIV_027930 | Syntenic |
| ROP18 | TGME49_005250 | VIIa | - | - |
| ROP19 | TGME49_042240 | VI | NCLIV_017440 | Syntenic |
| ROP20 | TGME49_058230 | VIIb | NCLIV_028170 | Syntenic |
| ROP21 | TGME49_063220 | VIIb | NCLIV_024700 | Syntenic |
| ROP22 | TGME49_007700 | Ib | NCLIV_002650 | Syntenic |
| ROP23 | TGME49_039600 | VI | NCLIV_016220 | Syntenic |
| ROP24 | TGME49_052360 | III | NCLIV_007450 | Syntenic |
| ROP25 | TGME49_002780 | VIIa | NCLIV_022130 | Syntenic |
| ROP26 | TGME49_011260 | IV | NCLIV_011730 | Syntenic |
| ROP27 | TGME49_113330 | XI | NCLIV_056620 | Syntenic |
| ROP28 | TGME49_058370 | VIIb | NCLIV_028130 | Syntenic |
| ROP29 | TGME49_042230 | VI | NCLIV_017430 | Syntenic |
| ROP30 | TGME49_027010 | X | NCLIV_046000 | Syntenic |
| ROP31 | TGME49_058800 | VIIb | NCLIV_027710 | Syntenic |
| ROP32 | TGME49_070920 | VIII | NCLIV_035860 | Syntenic |
| ROP33 | TGME49_001130 | VIIa | NCLIV_023260 | Syntenic |
| ROP34 | TGME49_040090 | VI | NCLIV_000650 | Ia |
| ROP35 | TGME49_104740 | VIIa | NCLIV_044410 | IX |
| ROP36 | TGME49_007610 | Ib | NCLIV_002580 | Syntenic |
| ROP37 | TGME49_094560 | Ia | NCLIV_001460 | Syntenic |
| ROP38 | TGME49_042110 | VI | NCLIV_017410 | Syntenic |
| ROP39 | TGME49_062050 | VIIb | NCLIV_028170 | Syntenic |
| ROP40 | TGME49_091960 | IX | NCLIV_012920 | Syntenic |
| ROP41 | TGME49_066100 | X | NCLIV_048060 | Syntenic |
| ROP42 | TGME49_009980 | Ib | - | - |
| ROP43 | TGME49_010090 | Ib | - | - |
| ROP44 | TGME49_010110 | Ib | NCLIV_004220 | Syntenic |
| ROP45 | TGME49_081790 | VIIa | NCLIV_023580 | Syntenic |
| ROP46 | TGME49_030470 | VIII | NCLIV_030990 | Syntenic |
| ROP47 | TGME49_042120 | VI | NCLIV_017420 | Syntenic |
| ROP48 | TGME49_115940 | XI | NCLIV_058560 | Syntenic |
| ROP49 | TGME49_052500 | VIIa | NCLIV_022270 | Syntenic |
| ROP50 | TGME49_022410 | II | NCLIV_005800 | Syntenic |
| ROP51 | - | - | NCLIV_068890 | Unk |
| ROP52 | - | - | NCLIV_069590 | Unk |
| ROP53 | - | - | NCLIV_068850 | Unk |
| ROP54 | - | III | NCLIV_008640 | III |
| ROP55 | - | VIII | NCLIV_031550 | VIII |
| SUB2 | TGME49_114500 | XI | NCLIV_057550 | Syntenic |
| Toxofilin | TGME49_014080 | X | NCLIV_051340 | Syntenic |
| Toxopain1 | TGME49_049670 | XII | NCLIV_069550 | Unk |
|  |  |  |  |  |
|  |  |  |  |  |
| **Microneme genes** | |  |  |  |
|  |  |  |  |  |
| MIC1 | TGME49_091890 | IX | NCLIV_043270 | Syntenic |
| MIC2 | TGME49_001780 | VIIa | NCLIV_022970 | Syntenic |
| M2AP | TGME49_014940 | X | NCLIV_051970 | Syntenic |
| MIC3 | TGME49_119560 | IV | NCLIV_010600 | Syntenic |
| MIC4 | TGME49_008030 | Ib | NCLIV_002940 | Syntenic |
| MIC5 | TGME49_077080 | XII | NCLIV_068520 | Syntenic |
| MIC6 | TGME49_018520 | XII | NCLIV_061760 | Syntenic |
| MIC7 | TGME49_061780 | VIIb | NCLIV_025710 | Syntenic |
| MIC8 | TGME49_045490 | XII | NCLIV_062770 | Syntenic |
| MIC8-like1/MIC8.2 | TGME49_086740 | V | NCLIV_013920 | Syntenic |
| MIC9 | TGME49_045490 | XII | NCLIV_062760 | Syntenic |
| MIC10 | TGME49_050710 | XII | NCLIV_066250 | Syntenic |
| MIC11 | TGME49_004530 | VIIa | NCLIV_020720 | Syntenic |
| MIC12 | TGME49_067680 | IX | NCLIV_069310 | Unk |
| MIC13/MCP2 | TGME49_060190 | VIIb | NCLIV_026810 | Syntenic |
| MIC14 | - | - | NCLIV_033690 | VIII |
| MIC15 | TGME49_044180 | VI | NCLIV_018780 | Syntenic |
| MIC16 | TGME49_115520 | XI | NCLIV_058210 | Syntenic |
| MIC17A | TGME49_000230 | VIII | NCLIV_038100 | Syntenic |
| MIC17B | TGME49_000240 | VIII | NCLIV_038110 | Syntenic |
| MIC17C | TGME49_000250 | VIII | NCLIV_038120 | Syntenic |
| MIC18 | TGME49_009920 | Ib | NCLIV_004170 | Syntenic |
| MIC19 | - | - | NCLIV_038320 | IX |
| MIC20 | TGME49_038210 | VI | NCLIV_015580 | Syntenic |
| MIC21 | TGME49_038220 | VI | NCLIV_015590 | Syntenic |
| MIC22 | TGME49_075790 | III | NCLIV_007140 | Syntenic |
| MIC23 | TGME49_115540 | XI | NCLIV_058230 | Syntenic |
| MIC24 | TGME49_054430 | III | NCLIV_008720 | Syntenic |
| MIC25 | TGME49_115550 | XI | NCLIV_058240 | Syntenic |
| SUB1 | TGME49_004050 | VIIa | NCLIV_021050 | Syntenic |
| AMA1 | TGME49_055260 | VIIb | NCLIV_028680 | Syntenic |
| AMA2 | TGME49_115730 | XI | NCLIV_058410 | Syntenic |
| AMA3 | TGME49_100130 | XII | NCLIV_064590 | Syntenic |
| MCP3 | TGME49_008740 | Ib | NCLIV_003260 | Syntenic |
| MCP4 | TGME49_008730 | Ib | NCLIV_003250 | Syntenic |
| MCP5 | - | - | NCLIV_066750 | XII |
| MCP6 | - | - | NCLIV_054450 | XI |
| MCP7 | - | - | NCLIV_054425 | XI |
|  |  |  |  |  |
|  |  |  |  |  |
| **Dense granule genes** | |  |  |  |
|  |  |  |  |  |
| GRA1 | TGME49_070250 | VIII | NCLIV_036400 | Syntenic |
| GRA2 | TGME49_027620 | X | NCLIV_045650 | Syntenic |
| GRA3 | TGME49_027280 | X | NCLIV_045870 | Syntenic |
| GRA4 | TGME49_110780 | XI | NCLIV_054830 | Syntenic |
| GRA5 | TGME49_086450 | V | NCLIV_014150 | Syntenic |
| GRA6 | TGME49_075440 | X | NCLIV_052880 | Syntenic |
| GRA7 | TGME49_003310 | VIIa | NCLIV_021640 | Syntenic |
| GRA8 | TGME49_054720 | III | NCLIV_008990 | Syntenic |
| GRA9 | TGME49_051540 | XII | NCLIV_066630 | Syntenic |
| GRA10 | TGME49_068900 | VIII | NCLIV_037450 | Syntenic |
| GRA11 | TGME49_012410 | IV | - | - |
| GRA12 | TGME49_075850 | III | - | - |
| GRA14 | TGME49_039740 | VI | NCLIV_016360 | Syntenic |
| NTPase I | TGME49_077240 | XII | NCLIV_068460 | XII but not syntenic |
| NTPase II | TGME49_077270 | XII | NCLIV_068400 | Syntenic |
| PI-1 | TGME49_008450 | Ib | NCLIV_003120 | Syntenic |
| PI-2 | TGME49_008430 | Ib | NCLIV_003100 | Syntenic |
|  |  |  |  |  |
|  |  |  |  |  |
| **AP2 transcription factors** | |  |  |  |
|  |  |  |  |  |
| AP2Ib-1 | TGME49_008020 | Ib | NCLIV_002930 | Syntenic |
| AP2III-1 | TGME49_052370 | III | NCLIV_007460 | Syntenic |
| AP2III-2 | TGME49_053380 | III | NCLIV_007810 | Syntenic |
| AP2III-3 | TGME49_099150 | III | NCLIV_009360 | Syntenic |
| AP2III-4 | TGME49_099020 | III | NCLIV_009480 | Syntenic |
| AP2IV-1 | TGME49_120700 | IV | NCLIV_009580 | Syntenic |
| AP2IV-2 | TGME49_120680 | IV | NCLIV_009600 | Syntenic |
| AP2IV-3 | TGME49_118610 | IV | NCLIV_010930 | Syntenic |
| AP2IV-4 | TGME49_118470 | IV | NCLIV_011080 | Syntenic |
| AP2IV-5 | TGME49_011720 | IV | NCLIV_011370 | Syntenic |
| AP2IX-1 | TGME49_067460 | IX | NCLIV_038720 | Syntenic |
| AP2IX-2 | TGME49_064590 | IX | NCLIV_040560 | Syntenic |
| AP2IX-3 | TGME49_064480 | IX | NCLIV_040560 | Syntenic |
| AP2IX-4 | TGME49_088950 | IX | NCLIV_041340 | Syntenic |
| AP2IX-5 | TGME49_089710 | IX | NCLIV_041960 | Syntenic |
| AP2IX-6 | TGME49_090180 | IX | NCLIV_042430 | Syntenic |
| AP2IX-7 | TGME49_090630 | IX | NCLIV_042620 | Syntenic |
| AP2IX-8 | TGME49_106000 | IX | NCLIV_070180 | Syntenic |
| AP2IX-9 | TGME49_106620 | IX | NCLIV_044800 | Syntenic |
| AP2V-1 | TGME49_020530 | V | NCLIV_012620 | Syntenic |
| AP2V-2 | TGME49_085890 | V | NCLIV_014510 | Syntenic |
| AP2VI-1 | TGME49_040460 | VI | NCLIV_016660 | Syntenic |
| AP2VI-2 | TGME49_040900 | VI | NCLIV_017050 | Syntenic |
| AP2VI-3 | TGME49_044510 | VI | NCLIV_019060 | Syntenic |
| AP2VIIa-1 | TGME49_080470 | VIIa | NCLIV_019680 | Syntenic |
| AP2VIIa-2 | TGME49_080460 | VIIa | NCLIV_019690 | Syntenic |
| AP2VIIa-3 | TGME49_005650 | VIIa | NCLIV_020020 | Syntenic |
| AP2VIIa-4 | TGME49_003710 | VIIa | NCLIV_021340 | Syntenic |
| AP2VIIa-5 | TGME49_003690 | VIIa | NCLIV_021360 | Syntenic |
| AP2VIIa-6 | TGME49_003050 | VIIa | NCLIV_021870 | Syntenic |
| AP2VIIa-7 | TGME49_002490 | VIIa | NCLIV_022440 | Syntenic |
| AP2VIIa-8 | TGME49_082210 | VIIa | NCLIV_023820 | Syntenic |
| AP2VIIa-9 | TGME49_082220 | VIIa | NCLIV_023830 | Syntenic |
| AP2VIIb-1 | TGME49_062420 | VIIb | NCLIV_025410 | Syntenic |
| AP2VIIb-2 | TGME49_062000 | VIIb | NCLIV_025610 | Syntenic |
| AP2VIIb-3 | TGME49_055220 | VIIb | NCLIV_028720 | Syntenic |
| AP2VIII-1 | TGME49_029370 | VIII | NCLIV_030300 | Syntenic |
| AP2VIII-2 | TGME49_033120 | VIII | NCLIV_032930 | Syntenic |
| AP2VIII-3 | TGME49_073660 | VIII | NCLIV_034030 | Syntenic |
| AP2VIII-4 | TGME49_072710 | VIII | NCLIV_034610 | Syntenic |
| AP2VIII-5 | TGME49_071200 | VIII | NCLIV_035650 | Syntenic |
| AP2VIII-6 | TGME49_071030 | VIII | NCLIV_035780 | Syntenic |
| AP2VIII-7 | TGME49_069010 | VIII | NCLIV_037340 | Syntenic |
| AP2X-1 | TGME49_027900 | X | NCLIV_045500 | Syntenic |
| AP2X-10 | TGME49_015340 | X | NCLIV_052260 | Syntenic |
| AP2X-11 | TGME49_015570 | X | NCLIV_052490 | Syntenic |
| AP2X-2 | TGME49_025110 | X | NCLIV_047580 | Syntenic |
| AP2X-3 | TGME49_024230 | X | NCLIV_048320 | Syntenic |
| AP2X-4 | TGME49_024050 | X | NCLIV_048470 | Syntenic |
| AP2X-5 | TGME49_037090 | X | NCLIV_050970 | Syntenic |
| AP2X-6 | TGME49_037440 | X | NCLIV_051170 | Syntenic |
| AP2X-7 | TGME49_014840 | X | NCLIV_051890 | Syntenic |
| AP2X-8 | TGME49_014960 | X | NCLIV_051990 | Syntenic |
| AP2X-9 | TGME49_015150 | X | NCLIV_052140 | Syntenic |
| AP2XI-1 | TGME49_109410 | XI | NCLIV_053760 | Syntenic |
| AP2XI-2 | TGME49_110900 | XI | NCLIV_054920 | Syntenic |
| AP2XI-3 | TGME49_110950 | XI | NCLIV_054950 | Syntenic |
| AP2XI-4 | TGME49_115760 | XI | NCLIV_058430 | Syntenic |
| AP2XI-5 | TGME49_016220 | XI | NCLIV_059950 | Syntenic |
| AP2XII-1 | TGME49_018960 | XII | NCLIV_061420 | Syntenic |
| AP2XII-2 | TGME49_017700 | XII | NCLIV_062490 | Syntenic |
| AP2XII-3 | TGME49_046660 | XII | NCLIV_063450 | Syntenic |
| AP2XII-4 | TGME49_047700 | XII | NCLIV_063920 | Syntenic |
| AP2XII-5 | TGME49_047730 | XII | NCLIV_063940 | Syntenic |
| AP2XII-6 | TGME49_049190 | XII | NCLIV_065400 | Syntenic |
| AP2XII-7 | TGME49_050070 | XII | NCLIV_066150 | Syntenic |
| AP2XII-8 | TGME49_050800 | XII | NCLIV_066340 | Syntenic |
| AP2XII-9 | TGME49_051740 | XII | NCLIV_066800 | Syntenic |
