# Supplementary material for: Comparative Genomics of the Apicomplexan Parasites Toxoplasma gondii and Neospora caninum: Coccidia Differing in Host Range and Transmission Strategy
Source: PLoS Pathog. 2012 Mar 22;8(3):e1002567. doi: 10.1371/journal.ppat.1002567 (PMC3310773; doi:10.1371/journal.ppat.1002567)
Supplement: Table S5 — RNAseq mapping statistics for N. caninum Liverpool tachyzoites. TZ = tachyzoite. Sequencing was performed using an Illumina GAII as described in methods. Raw reads for these runs are available from the European Nucleotide Archive (ENA; http://www.ebi.ac.uk/ena/) under the run id listed. In library names A and B refer to biological replicates for a particular timepoint derived from distinct cultures of parasites. (DOCX) [file ppat.1002567.s014.docx]

Supplementary Table 5. RNAseq mapping statistics for *N. caninum* Liverpool tachyzoites

TZ = tachyzoite. Sequencing was performed using an Illumina GAII as described in methods. Raw reads for these runs are available from the Eurpean Nucleotide Archive (ENA; http://www.ebi.ac.uk/ena/) under the run id listed. In library names A and B refer to biological replicates for a particular timepoint derived from distinct cultures of parasites.

| **Library** | **Nc Day 3 TZ A** | **Nc Day 3 TZ B** | **Nc Day 4 TZ A** | **Nc Day 4 TZ B** | **Nc Day 6 TZ A** | **Nc Day 6 TZ B** |
| --- | --- | --- | --- | --- | --- | --- |
| **ENA run id** | ERR029935 | ERR029938 | ERR029936 | ERR029939 | ERR029940 | ERR029937 |
| **# reads** | 31423926 | 27727940 | 24886354 | 33809856 | 31697076 | 30013512 |
| **Read length** | 76 | 76 | 76 | 76 | 76 | 76 |
| **Paired?** | Yes | Yes | Yes | Yes | Yes | Yes |
| **# reads mapped** | 28069956 | 26456502 | 23492948 | 32169867 | 30723514 | 29260033 |
| **% reads mapped** | 89 | 95 | 94 | 95 | 96 | 97 |
| **Perfect mapping reads** | 14342580 | 16123338 | 12062860 | 18252225 | 17905312 | 17506102 |
| **% perfectly mapped** | 45.64222815 | 58.14834423 | 48.47178498 | 45.64222815 | 45.64222815 | 45.64222815 |
| **Uniquely mapped reads** | 26943630 | 25279884 | 22379144 | 30553113 | 29362163 | 27984826 |
| **% uniquely mapped** | 85 | 91 | 89 | 90 | 92 | 93 |
| **Mapped to CDS** | 16444553 | 16552176 | 13411049 | 18862083 | 18222735 | 17580985 |
| **% mapped to CDS** | 58.58417804 | 62.56373575 | 57.0854241 | 58.6327665 | 59.31201424 | 60.08532184 |
