# Supplementary material for: Comparative Genomics of the Apicomplexan Parasites Toxoplasma gondii and Neospora caninum: Coccidia Differing in Host Range and Transmission Strategy
Source: PLoS Pathog. 2012 Mar 22;8(3):e1002567. doi: 10.1371/journal.ppat.1002567 (PMC3310773; doi:10.1371/journal.ppat.1002567)
Supplement: Table S6 — RNAseq mapping statistics for T. gondii VEG tachyzoites. T. gondii RNAseq experiments were performed using the VEG strains due to their amenity in the lab, while the Me49 genome was used for mapping due to its superior assembly and annotation. Example day three and four RNAseq runs were mapped to the T. gondii VEG strain genome sequence to determine whether mapping was significantly improved relative to Me49. Mapping was very similar in both cases suggesting that mapping VEG transcriptome data to Me49 was a reasonable approach. (DOCX) [file ppat.1002567.s015.docx]

Supplementary Table 6. RNAseq mapping statistics for *T. gondii* VEG tachyzoites

*T. gondii* RNAseq experiments were performed using the VEG strains due to their amenity in the lab, while the Me49 genome was used for mapping due to its superior assembly and annotation. Example day three and four RNAseq runs were mapped to the *T. gondii* VEG strain genome sequence to determine whether mapping was significantly improved relative to Me49. Mapping was very similar in both cases suggesting that mapping VEG transcriptome data to Me49 was a reasonable approach.

| **Library** | **Tg Day 3 TZ A** | **Tg Day 3 TZ B** | **Tg Day 3 TZ B** | **Tg Day 4 TZ A** | **Tg Day 4 TZ B** | **Tg Day 4 TZ B** | **Tg Day 6 TZ A** | **Tg Day 6 TZ B** |
| --- | --- | --- | --- | --- | --- | --- | --- | --- |
| **ENA run id** | ERR029947 | ERR029942 | ERR029942 | ERR029948 | ERR029941 | ERR029941 | ERR029945 | ERR029946 |
| **# reads** | 32814282 | 32424566 | 32424566 | 50123384 | 27865300 | 27865300 | 55649170 | 36211228 |
| **Read length** | 76 | 76 | 76 | 76 | 76 | 76 | 76 | 76 |
| **Paired?** | Yes | Yes | Yes | Yes | Yes | Yes | Yes | Yes |
| **Reference** | TGME49 | TGME49 | TGVEG | TGME49 | TGME49 | TGVEG | TGME49 | TGME49 |
| **# reads mapped** | 32048757 | 30937280 | 31062539 | 49207380 | 26786393 | 27314593 | 54193215 | 35344357 |
| **% reads mapped** | 97 | 95 | 95 | 98 | 96 | 98 | 97 | 97 |
| **Perfect mapping reads** | 13695855 | 10446437 | 14021898 | 21656764 | 9445540 | 12701881 | 22511129 | 15422916 |
| **% perfectly mapped** | 42 | 32 | 43.24467442 | 43 | 34 | 45.58314822 | 40 | 43 |
| **Uniquely mapped reads** | 30571301 | 29845604 | 29107467 | 46759332 | 25528618 | 24669624 | 52384702 | 34021465 |
| **% uniquely mapped** | 93 | 92 | 89 | 93 | 91 | 88 | 94 | 93 |
| **Mapped to CDS** | 17512930 | 16386827 | 15486436 | 26480266 | 13884160 | 13031639 | 29574619 | 19811541 |
| **% mapped to CDS** | 55 | 53 | 50 | 54 | 52 | 48 | 55 | 56 |
