# Supplementary material for: Comparative Genomics of the Apicomplexan Parasites Toxoplasma gondii and Neospora caninum: Coccidia Differing in Host Range and Transmission Strategy
Source: PLoS Pathog. 2012 Mar 22;8(3):e1002567. doi: 10.1371/journal.ppat.1002567 (PMC3310773; doi:10.1371/journal.ppat.1002567)
Supplement: Table S7 — Varieties of tRNA genes found in T. gondii Me40 and N. caninum Nc-Liv genomes. (DOCX) [file ppat.1002567.s016.docx]

Supplementary Table 7. Varieties of tRNA genes found in *T. gondii* Me40 and *N. caninum* Nc-Liv genomes

| codon | anticodon | amino acid | Neospora | Toxoplasma | codon | anticodon | amino acid | Neospora | Toxoplasma | codon | anticodon | amino acid | Neospora | Toxoplasma | codon | anticodon | amino acid | Neospora | Toxoplasma |
| --- | --- | --- | --- | --- | --- | --- | --- | --- | --- | --- | --- | --- | --- | --- | --- | --- | --- | --- | --- |
| TTT | AAA | Phe |  |  | TCT | AGA | Ser | 3 | 4 | TAT | ATA | Tyr |  |  | TGT | ACA | Cys |  |  |
| TTC | GAA | Phe | 4 | 5 | TCC | GGA | Ser |  |  | TAC | GTA | Tys | 3 | 4 | TGC | GCA | Cys | 4 | 5 |
| TTA | TAA | Leu | 1 | 1 | TCA | TGA | Ser | 2 | 2 | TAA | TTA | STP |  |  | TGA | TCA | STP | 1 | 1 |
| TTG | CAA | Leu | 2 | 2 | TCG | CGA | Ser | 2 | 2 | TAG | CTA | STP |  |  | TGG | CCA | Trp | 2 | 4 |
| CTT | AAG | Leu | 4 | 4 | CCT | AGG | Pro | 5 | 5 | CAT | ATG | His |  |  | CGT | ACG | Arg | 4 | 5 |
| CTC | GAG | Leu |  |  | CCC | GGG | Pro |  |  | CAC | GTG | His | 3 | 3 | CGC | GCG | Arg |  |  |
| CTA | TAG | Leu | 1 | 2 | CCA | TGG | Pro | 4 | 4 | CAA | TTG | Gln | 2 | 3 | CGA | TCG | Arg | 2 | 2 |
| CTG | CAG | Leu | 3 | 3 | CCG | CGG | Pro | 3 | 3 | CAG | CTG | Gln | 3 | 4 | CGG | CCG | Arg | 1 | 1 |
| ATT | AAT | Ile | 3 | 4 | ACT | AGT | Thr | 3 | 4 | AAT | ATT | Asn |  |  | AGT | ACT | Ser |  |  |
| ATC | GAT | Ile | 1 |  | ACC | GGT | Thr |  |  | AAC | GTT | Asn | 6 | 5 | AGC | GCT | Ser | 4 | 4 |
| ATA | TAT | Ile | 1 | 1 | ACA | TGT | Thr | 2 | 3 | AAA | TTT | Lys | 4 | 3 | AGA | TCT | Arg | 2 | 2 |
| ATG | CAT | Met | 6 | 7 | ACG | CGT | Thr | 2 | 2 | AAG | CTT | Lys | 6 | 6 | AGG | CCT | Arg | 2 | 2 |
| GTT | AAC | Val | 4 | 5 | GCT | AGC | Ala | 3 | 3 | GAT | ATC | Asp |  |  | GGT | ACC | Gly |  |  |
| GTC | GAC | Val |  |  | GCC | GGC | Ala |  |  | GAC | GTC | Asp | 6 | 6 | GGC | GCC | Gly | 5 | 6 |
| GTA | TAC | Val | 1 | 2 | GCA | TGC | Ala | 3 | 4 | GAA | TTC | Glu | 4 | 4 | GGA | TCC | Gly | 3 | 6 |
| GTG | CAC | Val | 3 | 3 | GCG | CGC | Ala | 3 | 3 | GAG | CTC | Glu | 5 | 6 | GGG | CCC | Gly | 2 | 2 |
