# Supplementary material for: Comparative Genomics of the Apicomplexan Parasites Toxoplasma gondii and Neospora caninum: Coccidia Differing in Host Range and Transmission Strategy
Source: PLoS Pathog. 2012 Mar 22;8(3):e1002567. doi: 10.1371/journal.ppat.1002567 (PMC3310773; doi:10.1371/journal.ppat.1002567)
Supplement: Text S1 — Putative early bradyzoite differentiation in N. caninum at day six of culture. (DOC) [file ppat.1002567.s017.doc]

## Text S1. Putative early bradyzoite differentiation in *N. caninum* at day six of culture

At day six of our *N. caninum* tachyzoite cultures we found changes in the transcriptional profiles of key genes involved in host-cell invasion, most notably in rhoptry genes and AP2 transcription factors (Figure S9). Further investigation identified down-regulation of well-characterised tachyzoite markers and up-regulation of bradyzoite markers. We reasoned therefore that our day six *N. caninum* parasites were beginning to convert to bradyzoites. SRS29B (SAG1), the canonical tachyzoite marker was reduced in expression by a factor of four, whilst BAG1 (a bradyzoite marker) increased by a factor of four from background levels. Tachyzoite-specific SRS genes SRS29C (SRS2) and SRS57 (SAG3) were also downregulated at day six whilst the bradyzoite-specific genes SAG4 and SRS13 were up-regulated. Dense granule genes GRA3, GRA4 and GRA8 were downregulated. MIC10, MIC1, MIC2, MIC6, MIC8, AMA1, SUB1 and other MIC homologues were downregulated, MIC12 was up-regulated.

We found a significant reduction in expression of rhoptry genes at day six. ROP1 was down by a factor of 17 from RPKM 5136 at day three to 295 at day six. A further 16 rhoptry genes were downregulated at day 6 (ROP4/7, ROP6, ROP14, ROP24, ROP40, ROP10, ROP14, ROP26, ROP39, ROP17, ROP12, ROP13, ROP9, ROP23, ROP15, ROP11). RON2, RON3, RON4 and RON5 were also downregulated. ROP33 was the only ROP gene up-regulated at day six. This finding may relate to a reduced need for invasion proteins during the quiescent bradyzoite stage.

Work in *T. gondii* has shown that bradyzoites shut down aerobic respiration in favour of anaerobic processes and the accumulation of amylopectin granules from the diversion of pyruvate into gluconeogenesis (Tomavo 2001). We see gene expression changes in day six *N. caninum* tachyzoites which suggest this may be occurring. Pyruvate dehydrogenase is reduced in expression, while lactate dehydrogenase, pyruvate carboxlase and PEP-carboxylase kinase increase in expression. This suggests a redirection of pyruvate from the TCA cycle into gluconeogenesis and the eventual accumulation of amylopectin as has been observed in *T. gondii* bradyzoites (Guerardel et al. 2005).

Stage-specific paralogues of respiratory enzymes are known in *Toxoplasma* and these are thought to be associated with the switch to anaerobic respiration. LDH1 and LDH2 are the tachyzoite and bradyzoite-specific isozymes respectively of lactate dehydrogenase. In *Neospora* we observed high levels of expression of NcLDH1 thoughout the tachyzoite stage. NcLDH2 was not expressed in days three or four but showed a significant increase in day six. There was consistently high expression of TgLDH1 and consistently low expression of TgLDH2 in all *Toxoplasma* timepoints. TgENO2 and TgENO1 are the tachyzoite and bradyzoite-specific isozymes of enolase. NcENO2 was found expressed at all timepoints, while NcENO1 was found not to be expressed at any timepoint.

Around 20% of genes over-expressed at day six encoded ribosomal proteins, relating to both 40S and 60S subunits. A further 12 overexpressed genes were involved in rRNA processing or binding functions. This indicates a concurrent or impending increase in translational activity, perhaps to translate bradyzoite-specific mRNAs. This finding highlights translational control in stage conversion as has been suggested by (Ellis et al. 2004).
