# Supplementary material for: Comparative Genomics of the Apicomplexan Parasites Toxoplasma gondii and Neospora caninum: Coccidia Differing in Host Range and Transmission Strategy
Source: PLoS Pathog. 2012 Mar 22;8(3):e1002567. doi: 10.1371/journal.ppat.1002567 (PMC3310773; doi:10.1371/journal.ppat.1002567)
Supplement: Text S2 — Identifying Non-coding RNA genes. (DOC) [file ppat.1002567.s018.doc]

## Text S2. Identifying Non-coding RNA genes

A large proportion of eukaryotic transcriptomes consists of non-coding RNAs (ncRNAs). Recent studies indicate that a large number of these non-coding structured RNAs play critical roles in regulating gene expression at multiple levels in diverse organisms (Jost et al.). Since non-coding RNAs (ncRNA) are known to be involved in the regulation of gene expression in other eukaryotes, we undertook a genome-wide search for known and novel ncRNAs by comparing the non-protein coding regions of *N. caninum* and *T. gondii* genomes with different ncRNA gene finding algorithms.

Figure S5A shows a flow chart describing our methodology for *de novo* ncRNA discovery. We identified 427 putative ncRNAs (groups). Several of these were found to be related to well-conserved RNAs of known function. Others appeared to be specific to Coccidia and one in particular was expanded in *N. caninum*. Due the nature of our approach, in which we identified elements by virtue of their conservation between *N. caninum* and *T. gondii*, we were unable to identify novel ncRNAs specific to either parasite.

The 427 groups were filtered on the basis of strong similarity to annotated genes, genes present in GenBank, and mitochondrial-like nuclear sequences earlier described in *T. gondii* [7]. Of the 196 groups filtered out, several had good matches to ncRNAs of known function and are described in the following sections.

**Methodology**

7912 intergenic sequences >100bp and 33466 intronic sequences >75bp were extracted from *T. gondii* Me49 genome sequence. 7149 such intergenic and 35090 intronic sequences were extracted from the *N. caninum* genome sequence. BLASTN was used to identify similarities between the *N. caninum* and *T. gondii* sequences (dust and seg filters invoked, exp value=1-E06). High-scoring segment pairs (HSPs) with either a minimum length of 50 bp and a pairwise identity of at least 80%, or a minimum length of 70 bp and pairwise identity of 70% were collected. This resulted in 20517 HSPs. These HSPs were then processed with 3 structure prediction programs; RNAz (Washietl et al. 2005), QRNA (Rivas and Eddy 2001) and Pfold (Knudsen and Hein 2003). The overlap in HSPs predicted to be structural RNAs by these 3 programs is shown in Figure S5B. Next, 512 HSPs predicted to be structural RNAs by at least 2 prediction algorithms and not overlapping known RNA genes in either genome were collected. HSPs with sequences exceeding 400 bp were split into 200 bp subsets overlapping by 50 bp and analysed individually. The complete HSP was further processed if any subset was predicted to be a structural RNA.

In each genome overlapping HSPs were merged into non-overlapping genomic loci and grouped between genomes: For example: if locus A in *T. gondii* participated in a HSP with locus B in *N. caninum* and in another HSP with locus C in *N. caninum*, loci A, B and C were grouped together. 427 such groups were formed.

**Identification of non-protein coding RNA genes with known function**

*Transfer-RNAs (tRNAs)*

Both genomes were searched for tRNA structures using tRNAscan-SE (Lowe and Eddy 1997). This resulted in 143 tRNA gene predictions in *N. caninum* and 162 in *T. gondii* (Table S7). This difference in absolute numbers does not reflect a systematic proliferation in one species; of all the tRNAs that are residing on assembled chromosomes, 119 are apparently shared between the two genomes, with 30 and 20 tRNAs being specific to *T. gondii* and *N. caninum*, respectively.

*Small nuclear RNAs (snRNAs)*

Spliceosomal snRNAs from another apicomplexan species, *Plasmodium falciparum*, were collected from the Ares Lab Malaria Browser [http://areslab.ucsc.edu/] (Chakrabarti et al. 2007), and *N. caninum* and *T. gondii* genomes were searched for similarity using WUBLAST. In both genomes, 3 copies of U1 RNA were identified, and 2 copies of both the U2 RNA and the U5 RNA. Both genomes contained a single U4 RNA, whereas the U6 RNA was identified once in *T. gondii* and twice in *N. caninum*.

*Ribosomal RNAs (rRNAs)*

The co-transcribed cluster of ribosomal RNAs is often absent in whole genome sequencing approaches due to their repetitive nature. The mono-cistronic 5S ribosomal RNA was detected in three copies in *N. caninum* (chrVIII:79608-79731, chrIX:4166786-4166909, and chrX:6085827-6085950). We found only one copy in *T. gondii* (chrIX:5137533-5137656), which appear to be flanked by genomic regions with high similarities to 18S and 28S ribosomal RNAs. This is consistent with the genomic layout of ribosomal RNAs in *T. gondii* reported previously (Gagnon et al. 1996).

*Putative snoRNAs*

A number of putative snoRNAs in both of the genomes were detected by sequence similarity. Group 95 had similarity to snoR20 CD-box snoRNA from *P. falciparum* (Chakrabarti et al. 2007), which has homologues in both yeast and human. By searching the Rfam database (Gardner et al. 2009) for annotated RNA genes, group 249 was predicted to be the U3 CD-box snoRNA.

Alternatively, the presence of specific snoRNA gene motifs may hint that the predicted RNA genes were in fact snoRNAs. The two groups, 63 and 396 resided within two different introns of the equivalent gene in both genomes (TGME49_013570 and NCLIV_069480; both with unknown function). Further, group 396 was present in two copies in *T. gondii*. Group 63 contained H/ACA snoRNA motifs and group 396 contained C/D-box snoRNA motifs.

*RNase MRP*

The MRP ribozyme is involved in ribosomal RNA processing. A scan against Rfam predicted group 351 to be a RNase MRP gene. The length of an RNase MRP gene is typically around 280 bp, whereas group 351 was over 900 bp. Interestingly, Rfam predicted another RNA gene, a U36 CD-box snoRNA upstream of RNase MRP within group 351.

*Signal Recognition Particle (SRP) RNA*

Group 424 showed slight similarity to the SRP RNA from *Plasmodium falciparum* (Chakrabarti et al. 2007) and is identical to the *T. gondii* SRP RNA from the SRP Database (Andersen et al. 2006). This gene was first computationally identified (Zwieb et al. 2005) and later transcription was confirmed experimentally (Chakrabarti et al. 2007). However, it has been noticed that the SRP gene overlaps a predicted open reading frame and may not be correctly predicted (Chakrabarti et al. 2007).

**Prediction of novel structured ncRNA Genes**

After removing sequence groups with similarity to known ncRNAs, any gene present in Genbank or mitochondrial-like nuclear sequences described in *T. gondii* (Ossorio et al. 1991), 231 sequence groups remained as candidates for novel, structured RNAs. 93.3% of all groups appeared to reside in synteny between the two genomes. Of the exceptions (14 groups), nine groups were situated on the same chromosomes (and all within similar regions as based on the coordinates) and two groups were located at unmapped contigs in at least one species. Only three groups had reasonable evidence for not being syntenically conserved (groups 91, 363 and 367).

Group 56 represented a family containing 11 sequences, three from *T. gondii* and eight from *N. caninum* (Figure S6). The three *T. gondii* sequences were situated as tandem repeats on chromosome X, whereas the *N. caninum* sequences stemmed from two tandem clusters each with four sequences, residing on chromosome X and an unmapped contig, respectively. The single *T. gondii* cluster was found between TGME49_027370, encoding an X-Pro dipeptidyl-peptidase domain-containing protein, and TGME49_027370, encoding a putative 60S ribosomal protein L3. The *N. caninum* cluster on chromosome X was found between genes NCLIV_045790 and NCLIV_045800.
