# Supplementary material for: Comparative Genomics of the Apicomplexan Parasites Toxoplasma gondii and Neospora caninum: Coccidia Differing in Host Range and Transmission Strategy
Source: PLoS Pathog. 2012 Mar 22;8(3):e1002567. doi: 10.1371/journal.ppat.1002567 (PMC3310773; doi:10.1371/journal.ppat.1002567)
Supplement: Text S3 — References for Tables S1, S2, S3, S4, S5, S6. (DOCX) [file ppat.1002567.s019.docx]

**Supplementary references**

1. Edgar RC (2004) MUSCLE: multiple sequence alignment with high accuracy and high throughput. Nucleic Acids Res 32: 1792-1797.

2. Waterhouse AM, Procter JB, Martin DM, Clamp M, Barton GJ (2009) Jalview Version 2--a multiple sequence alignment editor and analysis workbench. Bioinformatics 25: 1189-1191.

3. Tamura K, Dudley J, Nei M, Kumar S (2007) MEGA4: Molecular Evolutionary Genetics Analysis (MEGA) software version 4.0. Mol Biol Evol 24: 1596-1599.

4. Washietl S, Hofacker IL, Stadler PF (2005) Fast and reliable prediction of noncoding RNAs. Proc Natl Acad Sci U S A 102: 2454-2459.

5. Knudsen B, Hein J (2003) Pfold: RNA secondary structure prediction using stochastic context-free grammars. Nucleic Acids Res 31: 3423-3428.

6. Rivas E, Eddy SR (2001) Noncoding RNA gene detection using comparative sequence analysis. BMC Bioinformatics 2: 8.

7. Hofacker IL, Fekete M, Stadler PF (2002) Secondary structure prediction for aligned RNA sequences. J Mol Biol 319: 1059-1066.

8. Trapnell C, Pachter L, Salzberg SL (2009) TopHat: discovering splice junctions with RNA-Seq. Bioinformatics 25: 1105-1111.

9. Carver T, Berriman M, Tivey A, Patel C, Bohme U, et al. (2008) Artemis and ACT: viewing, annotating and comparing sequences stored in a relational database. Bioinformatics 24: 2672-2676.

10. Friedrich N, Santos JM, Liu Y, Palma AS, Leon E, et al. (2010) Members of a novel protein family containing microneme adhesive repeat domains act as sialic acid-binding lectins during host cell invasion by apicomplexan parasites. J Biol Chem 285: 2064-2076.

11. Barber J, Trees AJ, Owen M, Tennant B (1993) Isolation of Neospora caninum from a British dog. Vet Rec 133: 531-532.

12. Dubey JP, Hattel AL, Lindsay DS, Topper MJ (1988) Neonatal Neospora caninum infection in dogs: isolation of the causative agent and experimental transmission. J Am Vet Med Assoc 193: 1259-1263.

13. Davison HC, Trees AJ, Guy F, Otter A, Holt JJ, et al. (1997) Isolation of bovine Neospora in Britain. Vet Rec 141: 607.

14. Conrad PA, Barr BC, Sverlow KW, Anderson M, Daft B, et al. (1993) In vitro isolation and characterization of a Neospora sp. from aborted bovine foetuses. Parasitology 106 ( Pt 3): 239-249.

15. Yamane I, Kokuho T, Shimura K, Eto M, Haritani M, et al. (1996) In vitro isolation of a bovine Neospora in Japan. Vet Rec 138: 652.
